# Genome-Wide Analysis of Four Pathotypes of Wheat Rust Pathogen (*Puccinia graminis*) Reveals Structural Variations and Diversifying Selection

**DOI:** 10.3390/jof7090701

**Published:** 2021-08-27

**Authors:** Kanti Kiran, Hukam C. Rawal, Himanshu Dubey, Rajdeep Jaswal, Subhash C. Bhardwaj, Rupesh Deshmukh, Tilak Raj Sharma

**Affiliations:** 1 Pusa Campus, ICAR-National Institute for Plant Biotechnology, New Delhi 110012, India; kantikiran@rediffmail.com (K.K.); hukum.rawal@gmail.com (H.C.R.); Hemu.bt@gmail.com (H.D.); rajdeepjaswal52@gmail.com (R.J.); 2 Regional Station, ICAR-Indian Institute of Wheat and Barley Research, Shimla 171002, India; Subhash.Bhardwaj@icar.gov.in; 3 National Agri-Food Biotechnology Institute, Punjab 140306, India; rupesh0deshmukh@gmail.com; 4 Division of Crop Science, ICAR-Indian Council of Agricultural Research, New Delhi 110001, India

**Keywords:** *Puccinia graminis*, rust pathogens, genome sequencing, genome-wide comparative analysis, diversifying selection, genomic variations

## Abstract

Diseases caused by *Puccinia graminis* are some of the most devastating diseases of wheat. Extensive genomic understanding of the pathogen has proven helpful not only in understanding host- pathogen interaction but also in finding appropriate control measures. In the present study, whole-genome sequencing of four diverse *P. graminis* pathotypes was performed to understand the genetic variation and evolution. An average of 63.5 Gb of data per pathotype with about 100× average genomic coverage was achieved with 100-base paired-end sequencing performed with Illumina Hiseq 1000. Genome structural annotations collectively predicted 9273 functional proteins including ~583 extracellular secreted proteins. Approximately 7.4% of the genes showed similarity with the PHI database which is suggestive of their significance in pathogenesis. Genome-wide analysis demonstrated pathotype 117-6 as likely distinct and descended through a different lineage. The 3–6% more SNPs in the regulatory regions and 154 genes under positive selection with their orthologs and under negative selection in the other three pathotypes further supported pathotype 117-6 to be highly diverse in nature. The genomic information generated in the present study could serve as an important source for comparative genomic studies across the genus *Puccinia* and lead to better rust management in wheat.

## 1. Introduction

Rusts are among the most devastating fungal pathogens of wheat worldwide. Stem (*Puccinia graminis* f. sp. tritici), stripe (*P. striiformis* f. sp. tritici), and leaf (*P. triticina*) rust diseases of wheat have globally affected wheat production and food security [1,2]. All the rusts of wheat are known to occur in India, of which, stem (black) rust of wheat is restricted to about seven million hectares of peninsular and central India only. Because of its destructiveness and the economic significance of its cereal hosts, stem rust is one of the most widely studied among all plant rust pathogens [3]. Despite continuous genetic interventions to improve wheat yields, its production has been nearly stagnant during the last few years because of biotic and abiotic constraints. Since the biotrophic pathogens cannot be cultured on artificial medium, the molecular characterization of functional genes has been extremely difficult in the rust fungi [4]. Nevertheless, in the recent years, enormous amounts of genomic information of rust pathogens, generated through next generation sequencing (NGS) strategies [4,5,6,7,8,9,10,11,12] and other advanced technologies like genotyping-by-sequencing [13] have enabled the study of rust development and evolution and the variations in their virulence profiles. Wheat and rusts have co-existed and co-evolved hand-in-hand for centuries. Frequent evolution of existing pathotypes in wheat rusts renders the resistant varieties susceptible [14]. Generally, a wheat variety lasts for 4–5 years as new virulence evolves due to deletions and insertions in the existing pathotypes [15]. The emergence and spread of race TTKSK (Ug99) and its variants, having virulence for *Sr*31, *Sr*24 and *Sr*36 [16,17], and the very recently emerged new race TTRTF, first detected in Eritrea (2016) and southern Iran (2019), along with six isolates of race TTRTF have again posed a challenge to wheat production worldwide. Thus, the use of genomic tools to decipher signatures of diversifying selection in lineages on a genome-wide level [18,19,20,21] has served as one of the most vital strategies to be incorporated for future wheat improvement programs.

We sequenced whole genomes of four distinct pathotypes, GKBSC (14, 16G2), PTHSC (40A, 62G29), PTTSF (40-3, 127G29), and KRCSC (117-6, 37G19) of wheat stem rust (*P. graminis)* from India to investigate the genomic structural features and performed comparative analysis across the genomes. Hereafter, we have used the names 14, 40A, 40-3, and 117-6 for the pathotypes, respectively. Among these, pathotype 14 identified in 1959 is the least virulent and has not evolved over the years. Pathotype 40A identified in 1974has been predominant in India for more than 35 years. Pathotype 40-3 was identified in 2008 and is a more virulent form of 40A [22,23,24]. Pathotype 117-6 is the most virulent member of 117 group of pathotypes. It renders many of the durum wheat cultivars susceptible and follows a different lineage than 40A and 40-3 [25,26,27]. Studies on the inheritance of rust resistance in durum wheat have been limited compared to the bread wheat [28]. Host specificity has a major role for a successful infection of obligate pathogens like wheat rusts with additional intra-species host specificity of the rusts as a prominent characteristic. The degree of virulence of pathotypes 40A and 40-3 on durum wheat is significantly very low (22%) compared to bread wheat (52%). Contrastingly, pathotype 117-6 is most virulent (53%) on durum wheat and shows a very low degree (8%) of virulence on bread wheat. The degree of virulence on either of the wheat varieties has been reported to be very low (2–4%) for pathotype 14 [29]. Thus, a better understanding of the four pathotypes with unique features of selective host specificity between the two wheat genomes can be helpful for the development of varieties for long lasting resistance to stem rusts. The very first information of the *P. graminis* genome was for race SCCL of *P. graminis* by Duplessis et al. [8]. Recently the assembled genome of four individual isolates of an Australian pathotype 21-0 along with two other American *P. graminis* isolates were made available in the public domain [11,12]. Additionally, various studies like fitness potential of the pathogen, prevalence, and occurrence on differential hosts have been studied [3]. Todate, information on genomics of this pathogen from isolates of distinct pathotypes prevalent in India has not been reported. Therefore, the present study was conducted to decode the genomes of four strains of *P. graminis* to (i) generate high-quality draft genome sequences of four Indian pathotypes through whole genome sequencing, (ii) perform genome-wide comparative analyses among the four distinct pathotypes of *P. graminis,* and (iii) perform genome-wide diversification analyses of identified genes in the four pathotypes.

## 2. Materials and Methods

### 2.1. Materials

**Figure 1:**
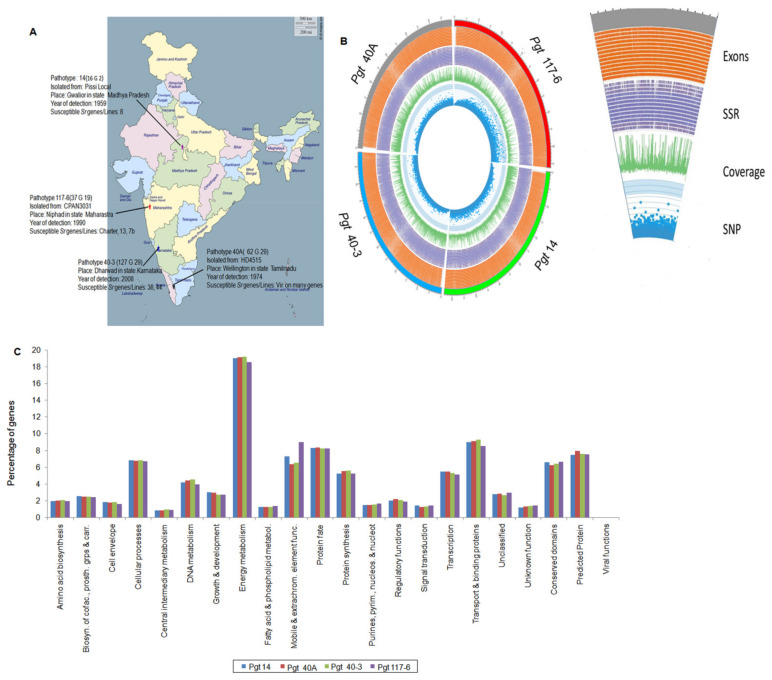
*P. graminis* pathotypes used in this study, various genomic features, and functional annotation of genes. (**A**) Description of the four pathotypes collected from various regions of India used in this study with their year of detection and host isolation. The map of India was downloaded from site (http://www.d-maps.com/carte.php?num_car=24868&lang=en, accessed on 12 July 2018) using “free-to-use images” which is under the license (Creative Commons—attribution 4.0 international CC BY 4.0) to be used as free for educational as well as commercial purposes. (**B**) CIRCOS plot of four individual *P. graminis* pathotypes. The outside of the outer-most circle in red, green, blue, and gray depicts the genome size of pathotypes 117-6, 14, 40-3 and 40A, respectively, with 1 Mb breakpoints increasing in the clockwise direction. Further inwards, the second circle is a density tile plot of all the annotated exons (orange color). The third inner circle in purple is the density histogram plot of total SSR content in the four pathotypes. The fourth circle is the density histogram plot depicting total genome coverage (green color). The inner-most circle in blue is the density scatter plot of total heterozygous SNPs identified in the four genomes. A clear distinction of SNP distribution in pathotype 117-6 can be observed as compared to the other three pathotypes. (**C**) Genes > 450bp excluding the hypothetical genes were annotated against NCBI nr database and categorized into 22 different functional categories. Percentage distribution of the genes in the individual class showed genes within energy metabolism to be the highest (~19%) in all the pathotypes, followed by mobile and extra chromosomal elements (~8%), and transport and binding proteins (~9%). About 7% genes were observed to fall under the categories of conserved and predicted proteins.

Four *P. graminis* f. sp. tritici pathotypes with distinct virulence profiles were selected for genome sequencing. The pathotypes presently are maintained (cryopreserved) at the Regional Station, Indian Institute of Wheat and Barley Research (Flowerdale, Shimla, India) (Figure 1A). Different sets of wheat differential hosts containing *Sr* genes (Supplementary Materials Tables S14 and S15) and their originators [30,31] were included to confirm the virulence profiles of the four pathotypes, viz. GKBSC (14, 16G2), PTHSC (40A, 62G29), PTTSF (40-3, 127G29), and KRCGC (117-6, 37G19) used in this study. The urediniospores of these pathotypes were multiplied on a susceptible wheat genotype ‘Agra local’ by a single spore infection.

### 2.2. Genomic DNA Isolation

Genomic DNA isolations were performed from the dikaryotic urediniospores of *P. graminis* pathotypes (14, 40A, 40-3, and 117-6) by using the CTAB method [32] with slight modifications [10].

### 2.3. Genome Sequence and Assembly

Individual separate paired-end (PE) libraries (100 bp) were prepared from the genomic DNA of all the pathotypes by using TruSeq DNA Library Preparation Kits as per the manufacture’s protocol and were sequenced using Hiseq 1000 (Illumina) automated sequencer (Illumina, Inc., San Diego, CA, USA). Reference-based assembly was performed for the processed data by GS Reference Mapper (Roche) version 2.5.3 with default parameters (minimum read length = 20 bp, minimum overlap length = 40 bp, minimum overlap identity = 90%, alignment identity score = 2, and all contig threshold = 100) using the genome sequence of *P. graminis* pathotype CDL 75-36-700-3 (Puccinia Group Sequencing Project, Broad Institute of Harvard and MIT (http://www.broadinstitute.org, accessed on 5 March 2017) as a reference. Unmapped reads were assembled and analyzed by using CLC Genomics Workbench 7.5.1. The quality of assembly was assessed by QUAST 3.2 software tool (Supplementary Materials Figure S1 and Table S1). Genome completeness of the assembled pathotypes was checked by CEGMA using a set of very highly conserved 248 core eukaryotic genes (CEGs), single-copy genes [33,34]. Raw reads of all four pathotypes were also mapped against the assembled data of self as well as against the other three pathotypes. The quality of the assembly was carried out by QUAST 3.2 software tool (Supplementary Materials Figure S1 and Table S1). Intra-pathotype SNPs were obtained by aligning the sequence reads of each pathotype to its assembled contigs. Further heterokaryotic and homokaryotic SNPs were predicted by aligning the reads of each isolate to the assembled contigs of the other pathotypes (inter-pathotype SNPs). Sequence data from this article have been deposited with the NCBI GenBank and their BioProject IDs (https://www.ncbi.nlm.nih.gov/bioproject/, accessed on 15 January 2017) under Accession No. LAQV00000000, LAQW00000000, LAQX00000000, LAQY00000000. If requested, the database will withhold release of data until publication.

### 2.4. Gene Prediction and Annotations

Genes were predicted from large contigs (≥2 kb) by FGENESH 3.1.2 (MolQuest2.2) trained against *Puccinia* spp. possessing no less than 80% homology with default parameters. For expression analysis, the predicted genes were searched against the National Center for Biotechnology Information (NCBI) EST dataset using the BLAST function. Functional annotation was done for the genes (≥450 bp) by searching against the NCBI-nr database, again via the BLAST function. Genes with significant hits (*E* ≤ 1 × 10^−10^) were categorized into different functional categories based on a literature search.

### 2.5. Identification of Repeat Elements within the Genomes of P. graminis Pathotypes

The repeat elements of various classes including long terminal repeats (LTRs), non-LTRs, and DNA transposon elements were identified in the assembled genomes against Repbase database (sp. Fungus) (http://www.girinst.org/repbase/update/, accessed on 3 October 2016) by MapRep module of MolQuest 2.2 with at least 80% homology.Full-length LTR elements were identified by using the LTR_FINDER software tool (http://tlife.fudan.edu.cn/ltr_finder/, accessed on 10 October 2016 [35]), with all specific parameters checked against the *Saccharomyces* repeat built-in database. Tandem repeat sequences were detected with the Tandem Repeats Finder 4 software with default parameters (https://tandem.bu.edu/trf/trf.html, accessed on 10 October 2016 [36]). Assembled sequences of the pathotypes were scanned for microsatellite repeats using the computer program MIcroSAtellite identification tool (MISA) (http://pgrc.ipk-gatersleben.de/misa/, accessed on 16 October 2016).

### 2.6. SNP and InDel Analysis

Whole-genome SNPs and InDels were detected using Sequence Alignment/Map tools (SAM tools) software package at 10× coverage with the quality value of Phred score ≥20. Sequence of *P. graminis* Race SCCL (CDL 36-700-3, Broad Institute, Cambridge, MA, USA) was used as a reference for the prediction of SNPs. The annotation of SNPs was performed using SnpEff software [37]. For the analysis of haplotype variations within the genomes, SNPs were detected by “Basic Variant Detection” module of CLC Genomics Workbench 7.5.1 with default parameters (Ploidy = 2, minimum coverage 10×, Variant Frequency ≥35%) by aligning raw reads of each pathotype against their respective contigs (assembled genome) for intra-pathotype SNPs and against assembled genomes of other three pathotypes for inter-pathotype SNPs. For depicting the heterozygosity level, inter-pathotype SNPs were classified as either ‘Homozygous’ or ‘Heterozygous’ having only one or more than one variant called at that position, respectively. Heterozygous SNPs, if referring to a variant position that is homozygous in other pathotypes, were classified as heterokaryotic SNPs, and Homozygous SNPs, if found polymorphic between two independent pathotypes, were classified as homokaryotic SNPs [6].

### 2.7. Secretome Analysis

Identification and analysis of the secretory proteins within the four *P. graminids* pathotypes was performed by using different software tools. Initially, proteins (≥50 amino acids) with a SignalP D-score = Y (SignalP version 4.1; www.cbs.dtu.dk/services/SignalP, accessed on 13 December 2017) and TargetPLoc = S (TargetP version1.1; www.cbs.dtu.dk/services/TargetP, accessed on 13 December 2017) were merged. These were then scanned for transmembrane spanning regions using TMHMM (TMHMM version2.0; http://www.cbs.dtu.dk/services/TMHMM, accessed on 15 December 2017). Peptides with 0 or 1 transmembrane regions were retained and transmembrane regions located in fewer than 10 amino acids in a mature peptide from a predicted cleavage site were considered for further analysis. The eventual locations of these proteins were predicted by the integral prediction of protein location score obtained by ProtComp version 10 (http://linux1.softberry.com/berry.phtml/berry.phtml?topic=protcompan&group=programssubgroup=proloc, accessed on 14 December 2017). Proteins showing the integral prediction of protein location and extracellular secreted (ES) were kept in the final secretome data set. BLASTP was used for the annotation of the predicted secretome. Conserved domains in the secretome were predicted through the Pfam domain database with profile gathering cut-off threshold [38]. Relative gene conservation of the total predicted ES proteins among the four pathotypes was determined using OrthoVenn web server (http://www.bioinfogenome.net/OrthoVenn/, accessed on 17 December 2017). Cysteine content in the extracellular secreted proteins was calculated and divided by the total number of residues in the peptide and converted to percentage. Potential pathogenicity genes in these genomes were identified by a BLAST search of predicted genes (≥450 bp) against 2647 protein sequences of PHI-base (Pathogen–Host Interactions database version 3.6) and genes with significant hits (with *E* ≤ 0.05 and bit score ≥100) were considered the pathogenicity-related genes.

### 2.8. Identification of Homologous Genes within and across the P. graminis Pathotypes

A combined approach of the ‘best bidirectional hit’ (BBH) method and the In-Paranoid program (INP) algorithm [39] with higher and more stringent threshold values was used for the identification of orthologs and paralogs [40,41]. Genes (≥450 bp) from each of the four genomes were searched against each other via a BLAST search following an all-against-all BLAST search. BLAST hits with bit score ≥100, E-value ≤ 0.05 and at least 40% identity between amino acid sequences over at least 70% of the protein length were filtered out as the significant hits [42,43]. Any two significant BLAST hits fulfilling the afore-mentioned 3-scale threshold (bit-score, E-value, and identity percentage) and those with bidirectional hits with each other were considered as paralogs or orthologs to each other based on if they belonged to the same or different pathotypes, respectively. The predicted ortholog pairs were clustered by using Excel sheets parsing into three groups with two, three, and four genes from two, three, and four different genomes, respectively. A circular diagram was constructed with Circos Table Viewer V0.63-9 [44], to show the syntenic view for one-to-one comparison of ortholog pairs having genes from any two pathotypes. To interactively show the number of ortholog pairs within two or three or all four isolates, a Venn diagram was constructed using VENNY 2.1 [45].

### 2.9. Phylogenetic Analysis

Comparative evolutionary analysis of the four pathotypes of *P. graminis*, along with the reference genome, CRL 75-36-700-3 (race SCCL) was performed through conservation distance matrix-based guide tree obtained by genome alignment using Mauve version 20150226 build 10 [46] with default parameters. For the SNP-based analysis, a phylogenetic tree of the pathotypes was constructed using ClustalX by applying the neighbor-joining method with 100 bootstrap replicates.

### 2.10. Diversifying Selection Analysis and Estimation of Substitution Rates

Among the four isolates, all genes (≥450 bp) with orthologs were chosen for the detection and analysis of diversifying selection. Gene sequences in each cluster were aligned by ClustalX 2.0 [47] and converted to PAML format by PAL2NAL version 14.0 [48]. For the estimation of synonymous and non-synonymous substitution rates, PAML format was used to calculate pairwise *dN/dS* ratios by YN00 of pamlX 1.3.1 [49]. Clusters with three or four genes and having atleast one gene with *dN/dS* ratios > 1 were further subjected to CODEML of pamlX 1.3.1 with two likelihood ratio tests (LRTs), i.e., model M1 (neutral) to model M2 (selection), and model M7 (beta) to M8 (beta and omega) to assess the site-specific diversifying selection. A gene was considered to be undergoing site-specific diversifying selection if both the M1/M2 and M7/M8 LRTs for that gene were found significant with the chi-square tests threshold, *p* < 0.05.

## 3. Results

### 3.1. Genome Sequencing and Assembly of the P. graminis Pathotypes

To investigate genetic variation between different pathotypes which are virulent/avirulent on standard differential genotypes (Figure 1A), four *P. graminis* f. sp. tritici (*P. graminis*) pathotypes from India (14, 40A, 40-3, and 117-6) were selected for sequencing using the NGS method. Approximately 6.2 Gb sequence data were generated for these pathotypes on the Illumina HiSeq 1000 platform. Reference-guided assembly using GS Reference Mapper Software (version 2.0, Roche) against the reference genome of *P. graminis* CDL 75-36-700-3, race SCCL [8] (Puccinia Group Sequencing Project, Broad Institute of Harvard and MIT (http://www.broadinstitute.org, accessed on 15 March 2017) resulted in a total of 58,140 to 70,264 sequence contigs with an N50 that ranged from 4.1 to 5.2 kb. We obtained 96× to 103× depth coverage of the assembled genome with the size varying from 59 to 66Mb (Table 1). The final assembled data were subjected to quality assessment by using QUAST 3.2 software tool (Supplementary Materials Figure S1 and Table S1) resulting in satisfactory scores of N50 and L50 for the pathotypes. The genome completeness of the four assembled pathotypes along with the reference genome was assessed with the Core Eukaryotic Gene Mapping Approach (CEGMA) with defined 248 single-copy conserved eukaryotic genes (CEGs). Results obtained in this study showed the presence of 75.81% to 79.84% of complete CEGs and 82.66% to 85.48% of partial CEGs in the genome assembly of all the pathotypes.

Normalized values for genome completeness with respect to the reference genome ranged from 84.30% to 88.79% for complete CEGs and from 89.13% to 92.17% for partial CEGs (Supplementary Materials Table S2A,B).

Genetic variations were expected between the two independent nuclei of asexual dikaryotic urediniospores. Inter- and intra-individual (within and across pathotypes) SNPs were predicted to investigate such variations in these four pathotypes. We identified an average SNP frequency of 23.75 ± 2.90 SNPs/kb between the two nuclei within a single individual (intra-pathotype SNPs) with highest frequency of 25.93 SNPs/kb for pathotype 40-3 followed by 25.71and 23.66 for pathotypes 40A and 14, respectively. A frequency of 19.68 SNPs/kb was obtained for the pathotype 117-6, being the lowest among the four pathotypes (Supplementary Materials Table S3). On average, heterokaryotic SNPs across the pathotypes were more frequent (11.78 ± 1.77 SNPs/kb) than homokaryotic SNPs (3.57 ± 2.35 SNPs/kb). The highest levels of diversity of over 13 SNPs/kb for heterokaryotic sites were found when reads of pathotypes 40A and 40-3 were individually mapped onto the other three pathotypes with an average of 13.18 ± 0.35 and 13.26 ± 0.34 SNPs/kb, respectively. Pathotype 117-6 was found to have the lowest diversity of 9.13 ± 0.23 SNPs/kb for heterokaryotic sites with the other three pathotypes whereas the diversity level for homokaryotic SNPs was the maximum, i.e., 5.32 ± 0.58 SNPs/kb with the other three pathotypes (Supplementary Materials Table S4).

### 3.2. Gene Prediction and Annotations

We predicted 13,854, 12,636, 12,670, and 15,401 protein-coding genes in the pathotypes 14, 40A, 40-3, and 117-6, respectively, by using FGENESH 3.1.2 (MolQuest2.2). Among the predicted proteins, an average of 90% of genes (≥150 bp) produced significant hits against NCBI database. Annotations of the genes with ≥450 bp (an average of 9273 genes in the four pathotypes) were performed by a BLASTP search against NCBI nr-database and 67% of these genes from the total predicted genes showed significant sequence similarity to the genes of all the four pathotypes in the database (Supplementary Materials Table S5). Among the twenty-three different functional classes assigned to the proteins, 46.4 to 49.3% of genes were hypothetical. The remaining 53.6 to 50.7% of the annotated genes in all the four pathotypes were categorized into twenty-two different functional classes (Figure 1C and Supplementary Materials Table S6). Four major classes with the genes “transport and binding” (8.9%), “predicted” (7.6%), “mobile and extra chromosomal elements” (7.3%), and “cellular processes” (6.7%) were obtained in all the four pathotypes. Genes (~19%) under the class “energy metabolism” showed illustrative differences in the pathotypes.

### 3.3. Identification and Analysis of Secretory Proteins

**Figure 2:**
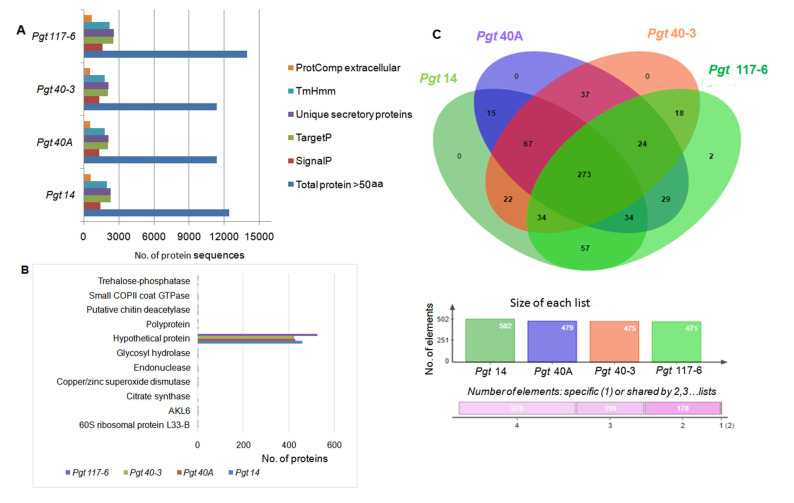
Genome-wide secretome analyses of the *P. graminis*pathotypes. (**A**) Identification of the extracellular secretory proteins was performed by subjecting the total predicted proteins (>50 aa) to various software tools and filtering processes. The exact number of protein sequences at the individual level of the filtering process is shown in the figure represented by different color bars. Details of the same can be seen in Supplementary Materials Table S7. (**B**) ABLAST search against the NCBI-nr database was performed in order to annotate the extracellular secreted proteins (ProtComp). Ten single-copy genes which were either present or absent in any of the four genomes were observed (Supplementary Materials Table S8). (**C**) Venn diagram represents the comparative analyses of extracellular secreted proteins performed for four pathotypes. None of the proteins showed specificity in genomes 14, 40A, or 40-3 with two proteins being specific to pathotype117-6. Color bars show orthology of genes within a genome with rest of the three genomes. Pink horizontal bar represents the number of genes orthologous between any two or three, or all the four genomes.

For successful infection, pathogenic fungi largely depend on a range of secreted proteins, particularly effectors. An in-house-designed pipeline was used to carry out the prediction of secreted proteins. All the four pathotype genomes encoded an average of 11.7% of the total predicted proteins (≥50 aa) as the potentially secreted proteins. The transmembrane proteins predicted through TMHMM were eliminated from the protein data set except for the proteins identified with value 1Tm. A total of 588 (14), 529 (40A), 535 (40-3), and 681 (117-6) proteins were predicted as extracellular secreted (ES) proteins using only mature peptide sequences (>20 aa). These ES proteins accounted for 4.75% of the predicted proteins in all the four pathotypes (Figure 2A and Supplementary Materials Table S7). Further, a BLAST search of these ES proteins against the NCBI-nr database produced significant hits for 468 (14), 435 (40A), 430 (40-3), and 532 (117-6) predicted secretory proteins. Among these hits, 79.6 to 82.2% of the proteins were annotated as hypothetical proteins, with only ten genes in pathotype 14, nine genes in pathotype 117-6, and seven genes each in pathotypes 40A and 40-3 with precise annotations and assigned functional classes. These were all single-copy genes within the genomes (Figure 2B). The remaining proteins (with no significant hits against NCBI nr-database) were further included in a BLAST search against the Australian isolate of *P. graminis,* pathotype 21-0 database (http://webapollo.bioinformatics.csiro.au/puccinia_graminis_tritici_PGTAus-pan/index.html, accessed on 18 December 2017). Analyses of the BLAST search against both the nr-database as well as the Australian isolate resulted in 13.8 to 16.8% of total secretory proteins with no significant hits. These putative proteins could thus be considered as novel proteins either specific or common among the four pathotypes. Conserved domains with precise function were searched with Pfam, and 7.6 to 10.4% of the ES proteins could be identified with a conserved functional domain in the respective genomes (Supplementary Materials Figure S2).

In order to determine the relative gene conservation among four *P. graminis* pathotypes, the putative ES effectors 588 (14), 529 (40A), 535 (40-3) and 681 (117-6) were searched for the presence of orthologs using the OrthoVenn webserver. Out of 612 clusters formed, 610 were orthologous clusters (gene clusters from any of the two pathotypes) and 270 were single-copy gene clusters which were shared single-copy genes among the four pathotypes. There were only two gene clusters unique to pathotype 117-6 with no homolog in the other three pathotypes. Individually, 502, 479, 475 and 471 gene clusters in the pathotypes 14, 40A, 40-3 and 117-6, respectively, shared orthologs with at least one of the pathotypes. While, 84, 50, 59 and 202 single-copy genes were unique to their respective genomes (Figure 2C). These gene clusters were further subjected to annotation analysis using GO and Swiss-prot databases and the majority of them did not show any hit with known genes.

### 3.4. Genome-Wide Analysis for Cysteine-Rich Genes

**Figure 3:**
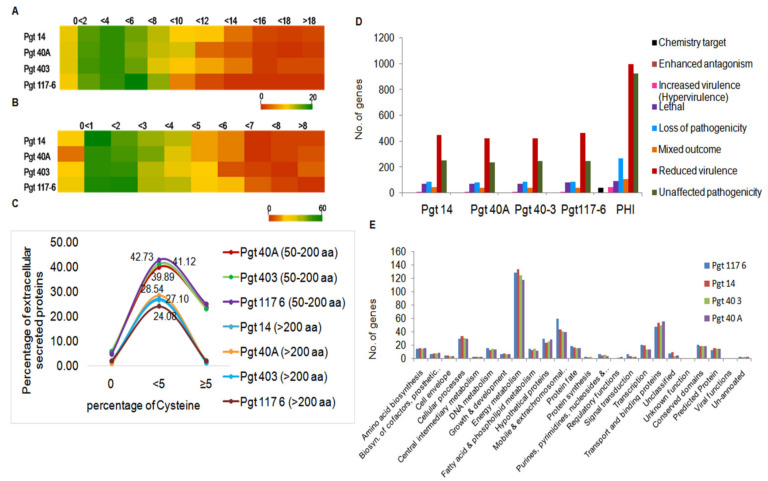
Cysteine-rich ES proteins and potential pathogenesis related proteins.(**A**) Heat map of small secretory proteins (50–200 aa) depicting a range (0 to >18) of cysteine residues in a percentage inversely proportional to the decreasing number of proteins. (**B**) Heat map of secretory proteins >200 aa showed most of the proteins (~18–52 in number) within the percentage of cysteine residues less than four. (**C**) Line chart of percentage of extracellular secreted proteins (ES) plotted against percentage of cysteine > and < than 5 within the four pathotypes. (**D**) All predicted genes (>150 aa) identified in the four pathotypes were subjected to PHI db genes to further categorize them based on their role in pathogenicity. Major group of genes were related to reduced virulence followed by other groups including unaffected pathogenicity, loss of pathogenicity, and lethal genes. (**E**) Functional annotation of the genes under reduced virulence into 23 classes showed energy metabolic genes as the most abundant class. Other major classes such as transport and binding, mobile and extra chromosomal elements, and cellular processes contained potential pathogenicity genes. A large number of genes were classified as hypothetical, conserved, predicted which could be novel or specific to individual pathotypes.

To investigate the cysteine-rich proteins within 588 (pathotype14), 529 (pathotype 40A), 535 (pathotype 40-3), and 681 (pathotype117-6) ES proteins identified in the pathotypes, further analysis was performed. In accordance to the growing evidence in the literature about effectors having unconventional characteristics, such as no predicted signal peptide, a low number of cysteine residues, or a large size [50,51,52,53], we performed our study by separating the proteins into sets of 50–200 aa and >200 aa categories. Considering both the sets, few proteins were observed to contain a very high percentage of cysteine residues (>8 to >18), while the majority of the proteins of four pathotypes had cysteine residues either ≥2 or ≤8. Comparatively the number of small ES proteins within pathotype 117-6 possessed a highest percentage of cysteine residues (Figure 3A,B). Interestingly, 23 to 25% of small ES proteins were found to contain more than 5% cysteine while only a negligible percentage contained more than 5% cysteine within the large ES proteins in the four pathotypes (Figure 3C). Overall our results revealed small ES proteins (50–200aa) to be prominently more rich in cysteine residues compared to the larger ES proteins (>200 aa) among all four pathotypes (Figure 3C).

### 3.5. Identification of Pathogenicity-Related Genes

Pathogenicity genes were determined for the genes >150 aa identified in the four pathotype genomes by using pathogen–host interaction (PHI) gene database version 3.6 [43]. The protein-coding genes (7.4 to 7.8%) identified in four *P. graminis* pathotypes showed homology with the genes present in the PHI db. Details of the number of genes sharing homology with the PHI db in the four *P. graminis* genomes analyzed in this study are given in Supplementary Materials Table S7. We obtained homologs to enhanced antagonism, loss of pathogenicity genes, lethal genes, increased virulence genes, mixed pathogenesis genes, and unaffected pathogenicity genes in the *P. graminis* genomes (Figure 3D).

Based on the functional groups identified earlier for all the predicted genes in this study, PHI gene homologs of “reduced virulence genes” were classified into 23 functional groups. Major groups included genes related to energy metabolism, mobile and extrachromosomal elements, cellular processes, transport and binding, transcription, and genes under hypothetical, unpredicted, and conserved domains (Figure 3E). Interestingly, of the total average 12,271 protein-coding genes (>150 aa) identified in all the four pathotypes, ~7.4% genes showed similarity with the PHI database (Supplementary Materials Table S8), which is suggestive of their significance in pathogenesis.

### 3.6. Genome-Wide Identification of SNPs

In order to investigate polymorphism in the four *P. graminis* pathotypes, DNA variants (SNPs, InDels) were predicted against the reference genome [7] at a 10× depth coverage. On average, a density of one SNP per 64 bp was observed in the four pathotypes. Overall, we identified 93% substitutions, 43% insertions, and 25% deletions in pathotype 117-6. Deletions within pathotypes 40A and 40-3 were 30% while in pathotype 14, 28% deletions were obtained (Table 2).

**Figure 4:**
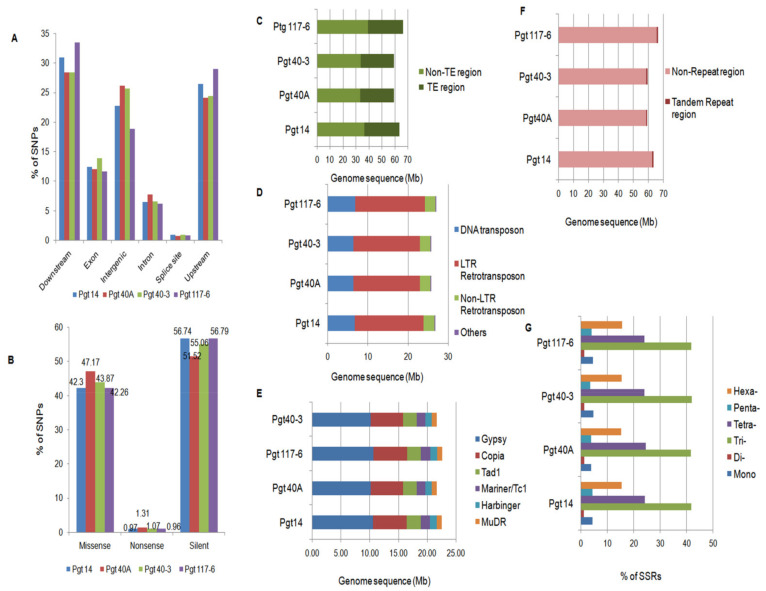
Whole-genome analysis of SNPs and repetitive content in the *P. graminis* pathotypes. (**A**) Whole genome identification of SNPs in different genomic regions of the pathotypes representing highest SNP percentages in the regulatory regions followed by the intergenic region in all the pathotypes. (**B**) Individual color bars representing the pathotypes with different types of SNPs within the exonic (coding) regions. Nonsense SNPs had the lowest percentage followed by the missense and silent SNPs in all the pathotypes. (**C**) Representation of dispersed repeats within the genome in terms of Mb containing transposable elements (TE) in all the pathotypes. (**D**) Categorization of TEs into three major classes (LTR, non LTR, and DNA transposons) and their content in the genome. (**E**) Identification of major families of class I and II elements occupying the genomes. (**F**,**G**) Representation of tandem repeats within the four genomes which are negligible compared to the TE space followed by percentage distribution of SSRs within the coding (CDS) regions of the pathotypes representing the tri-repeats as the most abundant followed by the tetra- and hexa- repeats, which was expected.

The type of effects (start codon gained or lost, stop codon gained or lost, exon deletion, or gene ablation due to deletion of a gene, etc.) caused by the variants (SNPs and InDels) across different genomic regions showed that most of these variants were found in the downstream and upstream regions (5kb upstream of the most distal transcription start site and 5 kb downstream of the most distal polyA addition site, respectively), with 28% and 24% of the total variations in the pathotypes 40A and 40-3, respectively. For variants in pathotypes 14 and 117-6, 31% and 33% were found in the downstream region and 26% and 30% in the upstream regions, respectively. The percentage of variants in the intergenic regions was more (26%) in pathotypes 40A and 40-3 than in pathotype 14 (23%) and pathotype 117-6 (19%). Intronic regions showed a similar percentage of variants in all the pathotypes (Figure 4A). Further, functional annotation of the SNPs within the exonic regions resulted in 43%, 48%, 45%, and 43%non-synonymous SNPs in the pathotypes 14, 40A, 40-3, and 117-6, respectively. Silent substitutions were most abundant with 57% in pathotypes 14 and 117-6, 55% in pathotype 40-3, and 52% in pathotype 40A (Figure 4B). On an average, the ratio of non-synonymous to synonymous SNPs was 0.82 in these four pathotypes.

### 3.7. Identification of Repetitive Elements

Total repeat contents were analyzed in the genome of the four pathotypes and were divided into two categories—like transposable elements (TEs) and tandem repeats. TEs were found to be 40.0% (~26 Mb) in the genomes (Table 1 and Figure 4C). The most dominant class was represented by the LTR retrotransposons with 27.3%, corresponding to ~17Mb of the genome in all the pathotypes. LTR retrotransposons were followed by DNA transposons and non-LTR retrotransposons with approximately 6.6 Mb (10.6%) and 2.6 Mb (4.2%) of their genomes, respectively (Figure 4D). These repeats were further annotated into different super families within the genomes of the pathotypes. The Gypsy (10.4 Mb) and Copia (5.6 Mb) elements were found to be the most abundant, followed by the presence of super families such as Tad 1, Tc1/Mariner, MuDR and Harbinger elements (~5 Mb) in their respective genomes (Figure 4E). Due to the lack of specific coding regions, certain elements (~4.0 Mb) could not be annotated into a specific super family and thus were placed into the class “Others”.

Only a negligible portion (~0.8Mb) of the assembled genomes (~1.3%) of all the four pathotypes were composed of tandem repeats (Figure 4F). Further analysis showed that the microsatellite repeat (SSR) distribution within the predicted protein coding region of pathotypes revealed tri-nucleotide repeats as the most abundant class, comprising 41.8%, followed by tetra- (24.5%) and hexa- (15.5%) repeats. Mono- and penta-repeats were both similar with ~4.0% occurrence within the genomes and the least represented class (1.3%) was the di-nucleotide repeats (Figure 4G). These results demonstrated a clear difference between the TEs and tandem repeat contents within the genomes of four pathotypes of *P. graminis,* which is a common feature among the genomes [54].

### 3.8. Conservation of Orthology and Paralogy Genes in the Pathotypes

**Figure 5:**
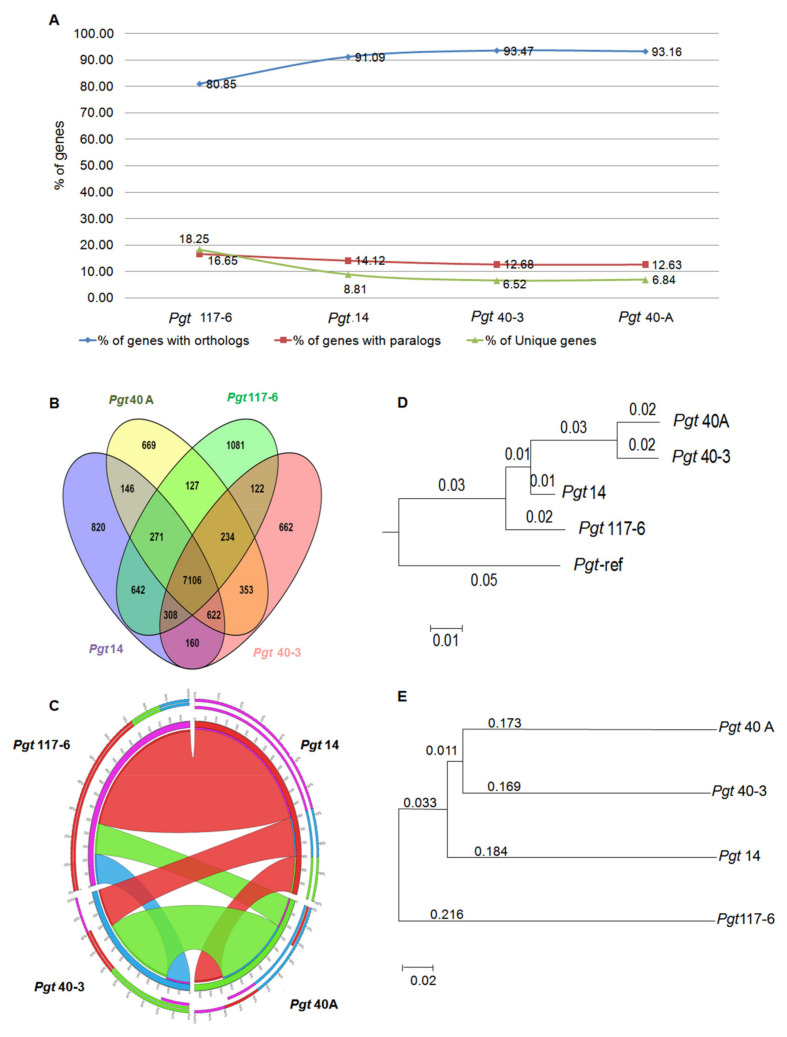
Genome-wide analyses of homologous genes and phylogenic analyses of the *P. graminis* pathotypes. (**A**) Percentage distribution of the homologous genes (paralogous and orthologous genes) within and across the genomes. (**B**) Venn diagram representation of the number of genes showing homology within (paralogous genes) and across (orthologous genes) genomes. (**C**) Synteny of one pathotype with any of the other three pathotypes. Colour green for Pgt 40A, colour blue for Pgt 40-3, colour red for Pgt 14 and colour violet for Pgt 117-6. (**D**) Guide tree obtained on the basis of complete genome alignment of the four *P. graminis* pathotypes. Branch lengths representing the conservation distance among the four pathotypes. (**E**) Molecular phylogenetic analysis by maximum likelihood method based on SNPs identified with 50% bootstraps cutoff value.

In order to investigate the protein evolution, we identified homologous genes among the four pathotypes (Figure 5A). A combined approach of the ‘best bidirectional hit’ (BBH) method and the In-Paranoid program (INP) algorithm [39] with higher and more stringent threshold values was used for the identification of orthologs and paralogs [40,41]. Using a BLASTp search, any two significant hits with bit score ≥ 100, at least 40% identity over 70% query coverage, and E-value 1 × 10^−20^ cut offs along with bidirectional hits with each other were considered as paralog pair being in the same pathotype or ortholog pair being in any of the other three pathotypes. 

Pairwise comparison of the numbers of orthologs between any two pathotypes in combination, demonstrated that while pathotypes 40A and 40-3 showed a good homology between each other with 353 conserved protein pairs, they shared the least number of pairs with pathotypes 117-6 and 14. Pathotype 14, in contrast to this, shared the highest number of homologous protein pairs with pathotype 117-6 (Supplementary Materials Table S9). These results indicate a distinction of pathotype 117-6 from pathotypes 40A and 40-3. Considering ortholog pairs among any three pathotypes at a time additionally demonstrated that pathotypes 117-6 and 14 are very distinct from pathotypes 40A and 40-3 (Supplementary Materials Table S9). All the pathotypes were observed with a higher percentage of orthologous gene pairs than unique and/or specific genes (Figure 5A). Comparative analysis of the homologous genes in all the four pathotypes suggested that 7106 homologs were present in all the pathotypes. These genes are referred to as core genes (Figure 5B). Interestingly, pathotype 117-6 had a fairly large number of unique and specific genes (1882), more than 3-fold higher than that of pathotypes 40A (593) and 40-3 (567) and more than 2-fold higher than pathotype 14 (830), further supporting its probable distinction from the other three pathotypes and indicative of its probable adaptive nature (Supplementary Materials Tables S3 and S9). Moreover, pathotype-specific genes having paralog pairs but no orthologous genes across pathotypes were highest in pathotype 117-6 (93) followed by only nine genes found in pathotype 14. There was a single gene specific to pathotype 40-3 while no such specific gene was identified in pathotype 40A (Supplementary Materials Table S9). Synteny analysis revealed 65% to 70% gene conservation between pathotypes 117-6 and 14 and 55% synteny among the genes of pathotypes 40-3 and 40A (Figure 5C). Altogether these results indicate the distant nature of pathotype 117-6 and pathotype 14 compared to pathotypes 40A and 40-3.

### 3.9. Evolutionary Relationship among the P. graminis Pathotypes

Phylogenetic analysis was performed to investigate the evolutionary relationship among the four pathotypes (40A, 40-3, 14, and 117-6) and the reference genome (CDL 75-36-700-3), based on whole-genome sequence alignment. The conservation distance calculated showed pathotypes 40A and 40-3 to be very closely related. Pathotype 14 was more closely related to pathotype 117-6 than 40A and 40-3 (Figure 5D). Similar results were observed in the analysis based on SNPs sharing a close relationship with pathotypes 40A and 40-3, and pathotype 117-6 was distantly related to three pathotypes (Figure 5E). These results showed the intraspecies discrimination of closely related pathotypes 40A and 40-3 with pathotype 117-6. 

### 3.10. Genome-Wide Analysis of Diversifying Selection

Apart from the effectors and pathogenicity-related genes in pathogens, numerous other genes have been known to undergo diversifying selection due to the potential strong selection pressure [55]. In order to investigate the evolutionary divergence, diversifying selection analysis was performed for all the predicted genes (≥450 bp (150 aa)) of the four pathotypes sharing orthologs among themselves. Signatures of diversifying selections were analyzed using two methods from PALM software [56]. Genes with at least one ortholog were analyzed by the counting method [57] utilizing YN00 to estimate the pairwise *dN/dS* ratios. Additionally, site-specific diversifying selection analysis for genes possessing at least two orthologs was performed by using two LRTs of CODEML. Maximum *dN/dS* ratios were obtained for pathotype 40A, followed by 40-3, and minimum in the pathotype 117-6. The site-specific diversifying selection was obtained for 5.1% of the genes in pathotype 14, 5.3% genes of pathotypes 40A and 40-3, and 4.9% genes of pathotype 117-6 (Supplementary Materials Table S10). Pathotype 117-6 had a greater number of lineage-specific genes that lacked the predicted orthologs among the pathotypes. Therefore, the percentage of genes analyzed for this pathotype was relatively low. The mean *dN/dS* ratio was 0.3 in pathotype 117-6, while the other three pathotypes (14, 40A, 40-3) were similar with the ratio of 0.28. 

### 3.11. Analysis of Substitution Rates of Sequence Divergence

**Figure 6:**
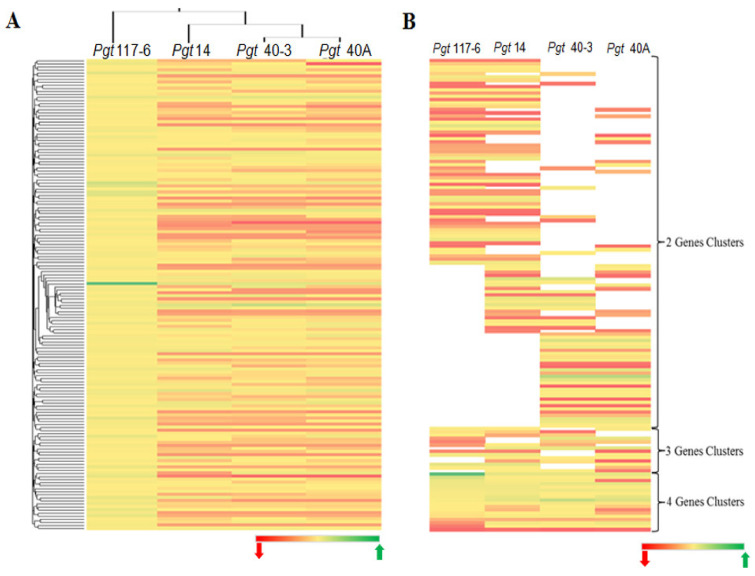
Comparative whole-genome diversifying analysis across all four pathotypes. (**A**) Clustered heat map of diversifying analysis of genes having orthology in all the four genomes. A set of 154 cDNA sequences in pathotype 117-6 with *dN/dS* values > 1 (only four pair clusters) was used as a base to sort out genes having orthologs in the other three pathotypes on the basis of *dN/dS* values < or >1. Green indicates higher (>1) *dN/dS* (w) and red corresponds to lower *dN/dS* (≤1) (w) value. (**B**) Clustered heat map generated for the genes having *dN/dS* values > 1 in all the four pathotypes. Clusters were sorted on the basis of orthologous pairs shared between any two (two way) pathotypes, any three (three way) pathotypes, and all four pathotypes (four way). Vacant (no color) space shows the absence of any shared genes in the respective pathotype.

Despite the *dN/dS* ratio varying among the gene pair combinations in all the pathotypes, the mean ratio was 0.30 ± 0.02, which was suggestive of a probable strong functional constrain for most of the genes. Among the genes analyzed for diversifying selection (YN00), we found that in all the four pathotypes around 97% of the genes had a *dN/dS* ratio <1. Genes with a*dN/dS* ratio = 1 were only found as single-copy genes in pathotypes 117-6 and 40A (Supplementary Materials Table S11). We further investigated the existence and conservation of the genes with a*dN/dS* ratio >1 by considering genes having orthology in the genomes of all the pathotypes. A set of these genes (154) only with *dN/dS* > 1 in pathotype 117-6 was used as a source to sort out the orthologs in the other three pathotypes inclusive of the genes with *dN/dS* < or > 1 (Figure 6A). This enabled the confirmation of the presence of orthologous genes along with their functional relationship, and if they are under some selection pressure with respect to pathotype 117-6. A comparative analysis was also performed to investigate the functional conservation among these genes (*dN/dS* > 1) within the genome of all the pathotypes. Clusters were generated on the basis of ortholog pairs shared between combinations of two pathotypes (two way), three pathotypes (three way), and all four pathotypes (four way). This analysis showed that pathotypes 117-6 and 14, and pathotypes 40A and 40-3 shared a higher number of genes common to each other as compared to any other two pathotypes observed together in the two-way gene cluster (Figure 6B). Functional annotation of all the genes (*dN/dS* *≥* 1) showed that approximately 75% of the genes were hypothetical in all the genomes analyzed in this study (Supplementary Materials Table S12). 

### 3.12. Identification of Genes under Site-Specific Diversifying Selection and Their Functional Categorization

Amino acid sequence analysis of genes (*p* < 0.05) underlying the selection in the present study suggested differences in the nature of the pressures exerted on them. Therefore, the site-specific diversifying genes (*p* < 0.05) with *dN/dS* ≥ 1 were further analyzed. Our results showed that some of the genes were under strong positive selection. Overall, the percentage of site-specific diversifying genes wascomparatively similar in pathotypes 14, 40-3, and 40A, while it waslowest in the pathotype 117-6 (Supplementary Materials Table S11). Distribution of these genes in different functional classes demonstrated that “energy metabolism”, “protein fate”, “mobile elements”, and “cellular process” were among the major categories that classified these genes (Supplementary Materials Table S13).

## 4. Discussion

It has been reported that about 85% of the global population requires wheat as one of their only calorie sources [58]. Wheat is cultivated in about 215 million hectares in the world and provides 20% of the calorie and protein requirements for 4.5 billion people in 94 countries [59]. Although, among the three existing rust diseases of wheat (leaf, stripe, and stem), stem rust is less common and prevalent compared to leaf rust but is considered the most destructive of the three wheat rust diseases [60]. Further, with the emergence of the strain TTKSK (Ug99) and the still-new race [61], the stem rust pathogen has recently acquired much attention due to the danger it poses to the global wheat productions in the near future. The mega wheat variety PBW343, covering nearly 8 million hectares in northern India has also become susceptible to Ug99 [62]. Therefore, a definite program to manage wheat rusts in India should be in place. The incidence and virulence pattern of stem rust pathogens are monitored on wheat crops in India for early detection of possible new virulence, evolution of pathotypes, and changes in pathotype composition and their distribution patterns in summer and regular wheat crops [23,24]. The pathotypes are designated as per the binomial system [63] with modifications [4]. The information thus generated can be used to select stem-rust-resistance genes for incorporation in the development and deployment of new wheat varieties to diversify resistance and avoid yield losses.

The years and places of detection of the four pathotypes selected and investigated in this study are quite different, but are being maintained at the national repository for studying their evolutionary update and disease prevalence each year. The phenotypic and phylogenetic features of the four pathotypes include pathotype 14, the least virulent in terms of not causing disease on wheat lines containing specific resistance genes, while pathotype 40-3 is the most recent and virulent, being able to overcome *Sr*7a, *Sr*13 and *Sr*30 (IT3+) genes. Pathotype 40A is avirulent to these three *Sr* genes but the virulence pattern for more than ~24 *Sr* genes is shared with pathotype 40-3. Pathotype 117-6 is virulent on durum wheat unlike the other three pathotypes which are virulent on bread wheat.Additionally, 117-6 is also virulent on *Sr*21(IT3+). Pathotype 117-6 is virulent on *Sr*13 but avirulent on *Sr*7a and *Sr*30(IT2), while pathotype 14 is avirulent on *Sr*7a, *Sr*13, and *Sr*30(IT 1-2) and virulent on *Sr*21(IT3+) (Supplementary Materials Figure S3 and Table S14). Thus, pathotype 14 could show an intermediary relationship with pathotypes 40A and 117-6in the evolutionary analysis. The phylogenetic analysis performed in this study based on the whole-genome comparisons as well as SNPs identified further pointed towards the diverse nature of the pathotypes.

The assembled genome data for the previously published *P. graminis* strain, CDL 75-36-700-3 (race SCCL) [8] from the U.S. confirms the high-quality of the assembly and larger (88.6 Mb) genome. Nevertheless, various quality assessments of our data indicate a relatively smaller size of the assembled genomes from India. The sequence depth coverage of 96× to 103× mapped reads of all the pathotypes added credence to our data. There was no evidence of whole-genome segmental duplications in the genomes (Supplementary Materials and Table S16) as similar to the strain CDL 75-36-700-3 from U.S. [8]. Since the dikaryotic spore stage was sequenced in the present study, the haplotype natures of the genomes were addressed by aligning the assembly against itself individually for each pathotype using CLC genomics workbench. Also, there is minimal possibility of any haplotype sequence being internally aligned elsewhere since the GS reference mapper allows a mapped read as a whole or a portion to be utilized only once during the whole-genome assembly while the possibility of repeats being assembled on top of each other could be considered. The total number of predicted CDS was found to be highest in pathotype 117-6 (15401) followed by pathotypes 14, 40A, and 40-3. The mean coding sequence length (1092 to 1152 bp) in all the four pathotypes was similar to the U.S. strain, i.e., 1075 bp [8]. With the whole-genome sequences of rust fungi now available [4,5,6,7,8,9,10], there are tremendous opportunities of deciphering the predicted genes and understanding their roles. In the present study, functionally annotated and predicted genes (~51.57%) containing significant (~19%) genes related to “energy metabolism”, and ~7–9% genes related to “cellular processes” and “transport and binding”, respectively, in the four individual *Puccinia* genomes, are a huge resource to understand the metabolic basis of the self-regulation within dormant/resting spores.

It has been demonstrated that diversifying selection has a strong impact on pathogens, especially in case of biotrophs, which have intimate connections to their hosts via effectors [64,65,66,67,68]. Species-specific effectors like *Melampsora lini* *Avr* gene homologs which are only found in *M. larici-populina*, as well as RTP1 effector homologs that are conserved across the rust fungi might have undergone natural selection [69,70]. This illustrates the existence of a triggered regulatory mechanism which encompasses a precise set of genes within the pathogen to survive and infect the host [71]. Therefore, a strong selection pressure is exerted on the genes. In the present study, comparative analysis of a core set of genes with *dN/dS* > 1 in pathotype 117-6 demonstrated that a majority of the orthologs present in the other three pathotypes (14, 40A, and 40-3) possess *dN/dS* < 1, possibly indicating a greater adaptability of pathotype 117-6 compared to other three pathotypes. Furthermore, orthologs of the genes under positive selection were predominantly shared between pathotypes 117-6 and 14 rather than with any other pathotype when compared individually. Therefore, the results of the present study indicate that pathotype 117-6 possesses lineage-specific genes which could be under strong positive selection. Also, a co-relation of an old avirulent pathotype (14) with a recent, virulent, and adaptable pathotype (117-6) could be seen, which suggests conservation of certain essential groups of genes. Thus, this study was focused on finding the impact of diversifying selection on the whole repertoire of predicted genes so that a common set of rust-specific genes could be identified within the different functional classes of these four pathotypes. Identification of such specific genes with unique features within individual pathotype would enable a better understanding of the evolution of recent virulent rust pathotypes which follow a different lineage.

Despite increasing evidence that not all the effectors are small in size and have cysteine-rich peptides, secreted pathogen proteins are still referred to as potential effectors [72]. Of the total ES proteins identified and analyzed in the present study, pathotype 117-6 showed 2 to 6% higher proteins with cysteine content >5 compared to the other three pathotypes. Two of these hypothetical genes were specific to pathotype 117-6. Similarly, the potential pathogenicity genes of pathotype 117-6 showed greater homology with the reduced virulence genes in PHI db (www.phibase.org, accessed on 13 December 2017 [43]) than the pathotypes 14, 40A, and 40-3. These results seem to differentiate pathotype 117-6 from rest of the three pathotypes in terms of the number of effectors and pathogenicity-related genes that may have important roles in contributing to it being the most virulent and specific to durum wheat. Prediction of ES proteins and identification of small proteins enriched in cysteine amino acids with the secretion signal form strong evidence for the presence of potential effectors in the *P. graminis* genome.

Pathotype 117-6 seemed to belong to a different group in this study, which is consistent with the published reports [25,73]. Mutational events are considered to be one of the main reasons for genetic variations in rust fungi [74,75,76] and might be responsible for the emergence of new virulent races [77,78]. At the same time, fewer molecular variations within the species have been observed [65,79]. Consistent with this, our results on whole-genome DNA polymorphism analysis within the four individuals showed quite a similarity in terms of percent distribution of SNPs in the genomic regions. Our results suggested most of the genes to be under purifying selection and 3% of genes potentially under positive selection which possibly could be responsible for the existing diversity among the *P. graminis* pathotypes. However, diversity among populations is also observed due to genetic drift, migration, and demographic events [80,81]. Furthermore, comparative analysis demonstrated the conservation of functional genes and a close relationship among themselves despite the diversity observed. Point mutations in non-protein-coding DNA sequences can also have functional consequences, particularly if they affect a regulatory element [82]. Consistent with this, an increased percentage of SNPs identified in the downstream, upstream, and intergenic regions compared to the exonic regions in all the four individuals could be of significant importance.

## 5. Conclusions

In this study we sequenced the genomes of four distinct *P. graminis* pathotypes (14, 40A, 40-3, and 117-6) and performed genome-wide comparative analyses. This is the first report using NGS for reference-based genome sequencing and genome-wide comparative analysis of four Indian *P. graminis* pathotypes. Pathotype 117-6 showed significant difference in possessing small ES proteins with high cysteine content, SNP distribution in the regulatory regions, genes of adaptability, and fairly large numbers of unique and specific genes. Whole-genome phylogenetic analysis revealed that there may be evolutionary relationships among the pathotypes in terms of host specificity, insertion and deletion events, and virulence profiles. Pathotype 14, being an older avirulent strain, could probably have an intermediary relationship between the bread wheat pathotypes, 40A and 40-3 and the durum wheat pathotype, 117-6. Considering any possible artifacts during sequencing and downstream analysis, this study nevertheless could serve as an important genomic resource for studying population structure and diversity analysis. It could also be helpful in monitoring the evolution of new variants of the rust pathogen and mapping genes for various traits to further enable a better management of the stem rust pathogen.

## Figures and Tables

**Table 1:** Sequencing and assembly statistics of the four *P. graminis* pathotypes.

*P.graminis* Pathotypes				
Parameters	14	40A	40-3	117-6
Input reads	67,098,012 (6.57 Gb)	61,276,376 (5.98 Gb)	60,290,992 (5.90 Gb)	65,648,262 (6.42 Gb)
Mapped Reads	62.77%	67.29%	67.26%	52.44%
Mapped Bases	62.00%	66.64%	66.42%	51.94%
Total contigs (assembled genome)	68,622 (63.31 Mb)	69,842 (59.15 Mb)	70,264 (59.25 Mb)	58,140 (66.25 Mb)
N50 (contigs) (bp)	4153	3573	3584	5288
Average contig length (bp)	922	846	843	1139
GC content of assembled genome (%)	43.61	43.64	43.64	43.58
Largest contig (bp)	65,288	43,631	39,779	46,544
Contigs ≥ 200 bases	37,103 (59.08 Mb)	36,874 (54.69 Mb)	36,934 (54.76 Mb)	33,359 (62.88 Mb)
Contigs ≥ 2Kb	7352 (42.14 Mb)	7364 (37.98 Mb)	7363 (38.07 Mb)	7499 (47.55 Mb)
Average contig length (>2 Kb) (bp)	5732	5157	5170	6341
N50 (>2 Kb contigs) (bp)	7152	6013	6022	8342
Depth coverage	103×	101×	99×	96×
Repeats (TE) (Mb)	26.84	25.76	25.78	27.05
Repeats (TE) % in assembled genome	42.40	43.56	43.51	40.83
Number of genes predicted	13,854	12,636	12,670	15,401
Mean gene length (bp)	1132.44	1151.94	1152.51	1092.92
Total number of exons	65,082	59,820	59,942	71,844
Mean number of exons per gene	4.69	4.73	4.73	4.66
Largest gene length (bp)	17,007	17,040	17,040	16,833
Genes (>150 bases)	12,424	11,347	11,371	13,943
Genes (>450 bases)	9419	8665	8694	10,315
Average gene length (bp) (>450 bases genes)	1561.15	1578.23	1578.19	1548.67
Mean number of exons per gene (>450 bases genes)	5.72	5.75	5.74	5.65

**Table 2:** Identification of SNPs and InDels in the *P. graminis* pathotype genomes.

Pathotypes	14	40A	40-3	117-6
SNPs	780,353	686,441	710,804	1,001,341
Insertions	51,662	44,403	47,055	46,206
Deletions	24,105	22,336	23,630	26,882

## Data Availability

Sequence data from this article have been deposited with the NCBI GenBank and their BioProject IDs (https://www.ncbi.nlm.nih.gov/bioproject/) under Accession No. LAQV00000000, LAQW00000000, LAQX00000000, LAQY00000000”. If requested, the database will withhold release of data until publication.

## Supplementary Materials

The following are available online at https://www.mdpi.com/article/10.3390/jof7090701/s1, Figure S1: Assembly statistics of Puccinia graminis pathotypes. Figure S2: Identification of conserved domains in the Puccinia graminis pathotypes. Figure S3: Depiction of IT (infection types) for the code of pathotypes on wheat leaves. Table S1: Genome assembly of the Puccinia graminispathotypes. Table S2: Statistics of the completeness of the genome based on analysis by CEGMA. Table S3: Identification of intra-pathotype genetic variation within the Puccinia graminispathotypes. Table S4: Identification of inter-pathotype genetic variations among the Puccinia graminis pathotypes. TableS5: Gene predictions of the Puccinia graminis pathotypes. Table S6: Categorization of annotated genes (>450) into various classes based on their functional roles. Table S7: Secretome analysis of the Puccinia graminis pathotypes. Table S8: Functional annotation of proteomes in Puccinia graminis pathotypes (against PHI database). Table S9: Analysis of pairwise orthologous genes across the Puccinia graminis pathotypes. Table S10: Diversifying selection analysis of genes (≥450bp) in four Puccinia graminis pathotypes. Table S11: Genes under specific selection pressure in the Puccinia graminis pathotypes. Table S12: Functional classification of genes with omega (dN/dS) > 1. Table S13: Functional classification of site-specific diversifying genes. Table S14: Virulence/avirulence formula for the Puccinia graminis pathotypes. Table S15: Wheat lines/varieties carrying Sr genes. Table S16: Segmental duplication in Puccinia graminis genomes.

## References

[1] Kolmer J.A. (2005). Tracking wheat rust on a continental scale. Curr. Opin. Plant Biol..

[2] Chen X. (2020). Pathogens which threaten food security: *Puccinia striiformis*, the wheat stripe rust pathogen. Food Secur..

[3] Kleinhofs A., Brueggeman R., Nirmala J., Zhang L., Mirlohi A., Druka A., Rostoks N., Steffenson B.J. (2009). Barley Stem Rust Resistance Genes: Structure and Function. Plant Genome.

[4] Zheng W., Huang L., Huang J., Wang X., Chen X., Zhao J., Guo J., Zhuang H., Qiu C., Liu J. (2013). High genome heterozygosity and endemic genetic recombination in the wheat stripe rust fungus. Nat. Commun..

[5] Cantu D., Govindarajulu M., Kozik A., Wang M., Chen X., Kojima K., Jurka J., Michelmore R.W., Dubcovsky J. (2011). Next Generation Sequencing Provides Rapid Access to the Genome of *Puccinia striiformis* f. sp. tritici, the Causal Agent of Wheat Stripe Rust. PLoS ONE.

[6] Cantu D., Segovia V., MacLean D., Bayles R., Chen X., Kamoun S., Dubcovsky J., Saunders D.G., Uauy C. (2013). Genome analyses of the wheat yellow (stripe) rust pathogen *Puccinia striiformis* f. sp. tritici reveal poly-morphic and haustorial expressed secreted proteins as candidate effectors. BMC Genom..

[7] Cuomo C.A., Bakkeren G., Khalil H.B., Panwar V., Joly D., Linning R., Sakthikumar S., Song X., Adiconis X., Fan L. (2017). Comparative Analysis Highlights Variable Genome Content of Wheat Rusts and Divergence of the Mating Loci. Genes Genomes Genet..

[8] Duplessis S., Cuomo C.A., Lin Y.-C., Aerts A., Tisserant E., Veneault-Fourrey C., Joly D., Hacquard S., Amselem J., Cantarel B.L. (2011). Obligate biotrophy features unraveled by the genomic analysis of rust fungi. Proc. Natl. Acad. Sci. USA.

[9] Kiran K., Rawal H.C., Dubey H., Jaswal R., Bhardwaj S.C., Prasad P., Pal D., Devanna B.N., Sharma T.R. (2017). Dissection of genomic features and variations of three pathotypes *of Puccinia striiformis* through whole genome sequencing. Sci. Rep..

[10] Kiran K., Rawal H.C., Dubey H., Jaswal R., Devanna B., Gupta D.K., Bhardwaj S.C., Prasad P., Pal D., Chhuneja P. (2016). Draft Genome of the Wheat Rust Pathogen (*Puccinia triticina*) Unravels Genome-Wide Structural Variations during Evolution. Genome Biol. Evol..

[11] Rutter W.B., Salcedo A., Akhunova A., He F., Wang S., Liang H., Bowden R.L., Akhunov E. (2017). Divergent and convergent modes of interaction between wheat and *Puccinia graminis* f. sp. tritici isolates revealed by the comparative gene co-expression network and genome analyses. BMC Genom..

[12] Upadhyaya N.M., Garnica D.P., Ekaraoglu H., Sperschneider J., Enemri A., Exu B., Mago R., Cuomo C., Rathjen J., Park R. (2015). Comparative genomics of Australian isolates of the wheat stem rust pathogen *Puccinia graminis* f. sp. tritici reveals extensive polymorphism in candidate effector genes. Front. Plant Sci..

[13] Edae E.A., Rouse M.N. (2020). Association mapping of resistance to emerging stem rust pathogen races in spring wheat using genotyping-by-sequencing. Plant Genome.

[14] Jain S.K., Prashar M., Bhardwaj S.C., Singh S.B., Sharma Y.P. (2009). Emergence of Virulence to Sr25 of *Puccinia graminis* f. sp. tritici on Wheat in India. Plant Dis..

[15] Bhardwaj S.C. (2013). Puccinia-Triticum interaction: An update. Indian Phytopath..

[16] Pretorius Z.A., Singh R.P., Wagoire W.W., Payne T.S. (2000). Detection of Virulence to Wheat Stem Rust Resistance Gene Sr31 in *Puccinia graminis* f. sp. tritici in Uganda. Plant Dis..

[17] Singh R.P., Hodson D.P., Huerta-Espino J., Jin Y., Bhavani S., Njau P., Herrera-Foessel S., Singh P., Singh S., Govindan V. (2011). The Emergence of Ug99 Races of the Stem Rust Fungus is a Threat to World Wheat Production. Annu. Rev. Phytopathol..

[18] Stukenbrock E.H., Jørgensen F.G., Zala M., Hansen T.T., McDonald B.A., Schierup M.H. (2010). Whole-Genome and Chromosome Evolution Associated with Host Adaptation and Speciation of the Wheat Pathogen Mycosphaerella graminicola. PLoS Genet..

[19] Raffaele S., Farrer R.A., Cano L.M., Studholme D.J., MacLean D., Thines M., Jiang R.H.Y., Zody M.C., Kunjeti S.G., Donofrio N.M. (2010). Genome Evolution Following Host Jumps in the Irish Potato Famine Pathogen Lineage. Science.

[20] Wicker T., Oberhaensli S., Parlange F., Buchmann J.P., Shatalina M., Roffler S., Ben-David R., Doležel J., Šimková H., Schulze-Lefert P. (2013). The wheat powdery mildew genome shows the unique evolution of an obligate biotroph. Nat. Genet..

[21] Sperschneider J., Ying H., Dodds P.N., Gardiner D.M., Upadhyaya N.M., Singh K.B., Manners J.M., Taylor J.M. (2014). Diversifying selection in the wheat stem rust fungus acts predominantly on pathogen-associated gene families and reveals candidate effectors. Front. Plant Sci..

[22] Bhardwaj S.C. (2011). Resistance Genes and Adult Plant Rust Resistance of Released Wheat Varieties of India.

[23] Prasad P., Bhardwaj S.C., Savadi S., Kashyap P.L., Gangwar O.P., Khan H., Singh S.B., Kumar S. (2018). Population distribution and differentiation of Puccinia graministritici detected in the Indian subcontinent during 2009–2015. Crop Protect..

[24] Sharma A.K., Saharan M., Bhardwaj S.C., Prashar M., Chatrath R., Tiwari V., Singh M., Sharma I. (2015). Evaluation of wheat (*Triticum aestivum*) germplasm and varieties against stem rust (Puccinia graminis f. sp. tritici) pathotype Ug99 and its variants. Indian Phytopathol..

[25] Jain S.K., Bhardwaj S.C., Prashar M., Singh S.B. (2013). Physiologic specialization and new virulences of *Puccinia graminis* f. sp. tritici causing black rust of wheat (*Triticum aestivum*) in India during 2005–2009. Indian J. Agric. Sci..

[26] Bhardwaj S.C., Prashar M., Kumar S., Datta D. (2006). Virulence and diversity of *Puccinia triticina* on wheat in India during 2002–2004. Indian J. Agric. Sci..

[27] Sai Prasad S.V., Singh S.K., Ambati V.D., Prakasha T.L., Singh J.B., Dubey V.G., Kantwa S.R., Mishra A.N. (2014). Introgression of stem rust resistance gene Sr36 into durum wheat back ground using marker assisted backcross breeding. J. Wheat Res..

[28] Mishra A.N., Kaushal K., Dubey V.G., Prasad S.V.S. (2014). New sources of stem rust resistance in durum wheat. Indian Phytopathol..

[29] Mishra A.N., Shirsekar G.S., Yadav S.R., Dubey V.G., Kaushal K., Prasad S.V.S., Pandey H.N. (2009). Protocols for evaluating resistance to leaf and stem rusts in durum and bread wheats. Indian Phytopathol..

[30] Bahadur P., Nagarajan S., Nayar S.K. (1985). A proposed system for virulence designation in India. 2. *Puccinia graminis* f sptritici. Proc. Plant Sci..

[31] Bhardwaj S.C., Gangwar O.P., Singh S.B., Saharan M.S., Sharma S. (2012). Rust situation and pathotypes of Puccinia species in Leh Ladakh in relation to recurrence of wheat rusts in India. Indian Phytopathol..

[32] Roose-Amsaleg C., De Vallavieille-Pope C., Brygoo Y., Levis C. (2002). Characterisation of a length polymorphism in the two intergenic spacers of ribosomal RNA in *Puccinia striiformis* f. sp. tritici, the causal agent of wheat yellow rust. Mycol. Res..

[33] Parra G., Bradnam K., Korf I. (2007). CEGMA: A pipeline to accurately annotate core genes in eukaryotic genomes. Bioinformatics.

[34] Parra G., Bradnam K., Ning Z., Keane T., Korf I. (2008). Assessing the gene space in draft genomes. Nucleic Acids Res..

[35] Xu Z., Wang H. (2007). LTR_FINDER: An efficient tool for the prediction of full-length LTR retrotransposons. Nucleic Acids Res..

[36] Benson G. (1999). Tandem repeats finder: A program to analyze DNA sequences. Nucleic Acids Res..

[37] Cingolani P., Platts A., Wang L.L., Coon M., Nguyen T., Wang L., Land S.J., Lu X., Ruden D.M. (2012). A program for annotating and predicting the effects of single nucleotide polymorphisms, SnpEff: SNPs in the genome of Drosophila melanogaster strain w1118; iso-2; iso-3. Fly.

[38] Finn R.D., Coggill P., Eberhardt R.Y., Eddy S.R., Mistry J., Mitchell A.L., Potter S.C., Punta M., Qureshi M., Sangrador-Vegas A. (2016). The Pfam protein families database: Towards a more sustainable future. Nucleic Acids Res..

[39] Hulsen T., Huynen M.A., De Vlieg J., Groenen P.M.A. (2006). Bench marking ortholog identification methods using functional genomics data. Genome Biol..

[40] Heidel A.J., Lawal H.M., Felder M., Schilde C., Helps N.R., Tunggal B., Rivero F., John U., Schleicher M., Eichinger L. (2011). Phylogeny-wide analysis of social amoeba genomes highlights ancient origins for complex intercellular communication. Genome Res..

[41] Rawal H.C., Singh N.K., Sharma T.R. (2013). Conservation, divergence, and genome-wide distribution of PAL and POX A gene families in plants. Int. J. Genom..

[42] Winnenburg R., Baldwin T.K., Urban M., Rawlings C., Köhler J., Hammond-Kosack K.E. (2006). PHI-base: A new database for pathogen host interactions. Nucleic Acids Res..

[43] Baugh L., Gallagher L.A., Patrapuvich R., Clifton M.C., Gardberg A.S., Edwards T.E., Armour B., Begley D.W., Dieterich S.H., Dranow D.M. (2013). Combining Functional and Structural Genomics to Sample the Essential Burkholderia Structome. PLoS ONE.

[44] Krzywinski M., Schein J., Birol I., Connors J., Gascoyne R., Horsman D., Jones S., Marra M.A. (2009). Circos: An information aesthetic for comparative genomics. Genome Res..

[45] Oliveros J.C., VENNY An Interactive Tool for Comparing Lists with Venn Diagrams. https://bioinfogp.cnb.csic.es/tools/venny/index.html.

[46] Darling A.C.E., Mau B., Blattner F.R., Perna N.T. (2004). Mauve: Multiple Alignment of Conserved Genomic Sequence with Rearrangements. Genome Res..

[47] Larkin M.A., Blackshields G., Brown N.P., Chenna R., McGettigan P.A., McWilliam H., Valentin F., Wallace I., Wilm A., Lopez R. (2007). Clustal W and Clustal X version 2.0. Bioinformatics.

[48] Suyama M., Torrents D., Bork P. (2006). PAL2NAL: Robust conversion of protein sequence alignments into the corresponding codon alignments. Nucleic Acids Res..

[49] Xu B., Yang Z. (2013). PAMLX: A Graphical User Interface for PAML. Mol. Biol. Evol..

[50] Djamei A., Kahmann R. (2012). Ustilago maydis: Dissecting the Molecular Interface between Pathogen and Plant. PLoS Pathog..

[51] Feng F., Yang F., Rong W., Wu X., Zhang J., Chen S., He C., Zhou J.-M. (2012). A *Xanthomonas uridine* 5’-monophosphate tra nsferase inhibits plant immune kinases. Nature.

[52] Houterman P.M., Speijer D., Dekker H.L., de Koster C.G., Cornelissen B.J.C., Rep M. (2007). The mixed xylem sap proteome of *Fusarium oxysporum*-infected tomato plants X veMol. Plant Pathol..

[53] Ridout C., Skamnioti P., Porritt O., Sacristan S., Jones J., Brown J.K. (2006). Multiple Avirulence Paralogues in Cereal Powdery Mildew Fungi May Contribute to Parasite Fitness and Defeat of Plant Resistance. Plant Cell.

[54] Lerat E. (2010). Identifying repeats and transposable elements in sequenced genomes: How to find your way through the dense forest of programs. Heredity.

[55] Gladieux P., Ropars J., Badouin H., Branca A., Aguileta G., de Vienne D., De La Vega R.C.R., Branco S.M., Giraud T. (2014). Fungal evolutionary genomics provides insight into the mechanisms of adaptive divergence in eukaryotes. Mol. Ecol..

[56] Yang Z. (2007). PAML 4: Phylogenetic Analysis by Maximum Likelihood. Mol. Biol. Evol..

[57] Yang Z., Nielsen R. (2000). Estimating Synonymous and Nonsynonymous Substitution Rates Under Realistic Evolutionary Models. Mol. Biol. Evol..

[58] Chaves M., Martinelli J.A., Wesp-Guterres C., Graichen F.A.S., Brammer S.P., Scagliusi S.M., Da Silva P.R., Wiethölter P., Torres G.A.M., Yamazaki-Lau E. (2013). The importance for food security of maintaining rust resistance in wheat. Food Secur..

[59] Braun H.J., Atlin G., Payne T. (2011). Multi-location testing as a tool to identify plant response to global climate change. Clim. Chang. Crop Prod..

[60] Sheikh F.A., Dar Z.A., Sofi P.A., Lone A.A., Shiekh N.A. (2017). Stem Rust of Wheat—A Basic Review. Int. J. Pure App. Biosci..

[61] Patpour M., Justesen A.F., Tecle A.W., Yazdani M., Yasaie M., Hovmøller M.S. (2020). First Report of Race TTRTF of Wheat Stem Rust (*Puccinia graminis* f. sp. tritici) in Eritrea. Plant Dis..

[62] Bhardwaj S.C., Prashar M., Prasad P. (2014). Ug99-Future Challenges. Future Challenges in Crop Protection against Fungal Pathogens.

[63] Bhardwaj S.C., Singh S.S., Hanchinal R.R., Singh G., Sharma R.K., Saharan M.S., Sharma I. (2012). Wheat rust pathotypes in Indian subcontinent then and now. Wheat-Productivity Enhancement under Changing Climate.

[64] Sperschneider J., Dodds P., Gardiner D., Manners J.M., Singh K., Taylor J. (2015). Advances and Challenges in Computational Prediction of Effectors from Plant Pathogenic Fungi. PLoS Pathog..

[65] Persoons A., Morin E., Delaruelle C., Payen T., Halkett F., Frey P., De Mita S., Duplessis S. (2014). Patterns of genomic variation in the poplar rust fungus Melampsora larici-populina identify pathogenesis-related factors. Front. Plant Sci..

[66] Kemen A.C., Agler M.T., Kemen E. (2015). Host–microbe and microbe–microbe interactions in the evolution of obligate plant parasitism. New Phytol..

[67] Gauthier N.W., Maruthachalam K., Subbarao K.V., Brown M., Xiao Y., Robertson C.L., Schneider R.W. (2014). Mycoparasitism of Phakopsora pachyrhizi, the soybean rust pathogen, by Simplicillium lanosoniveum. Biol. Control..

[68] Giraldo M., Valent B. (2013). Filamentous plant pathogen effectors in action. Nat. Rev. Genet..

[69] Fernandez D., Tisserant E., Talhinhas P., Azinheira H., Vieira A., Petitot A., Loureiro A., Poulain J., Da Silva C., Silva M.C. (2011). 454-pyrosequencing of Coffea arabica leaves infected by the rust fungus Hemileia vastatrix reveals in planta-expressed pathogen-secreted proteins and plant functions in a late compatible plant-rust interaction. Mol. Plant Pathol..

[70] Pretsch K., Kemen A., Kemen E., Geiger M., Mendgen K., Voegele R. (2012). The rust transferred proteins-a new family of effector proteins exhibiting protease inhibitor function. Mol. Plant Pathol..

[71] Flor H.H. (1971). Current Status of the Gene-For-Gene Concept. Annu. Rev. Phytopathol..

[72] Wang D., Tian L., Zhang D., Song J., Song S., Yin C., Zhou L., Liu Y., Wang B., Kong Z. (2020). Functional analyses of small secreted cysteine rich proteins identified candidate effectors in *Verticillium dahliae*. Mol. Plant Pathol..

[73] Singh S.K., Prakasha T.L., Divya A., Kantwa S.L., Prasad S.V.S., Mishra A.N. (2013). Evaluation of Indian durum wheat germplasm for the presence of Sr36 gene for resistance to pathotypes of stem rust race 117-group. Indian Phytopathol..

[74] Ellis J.G., Lagudah E., Spielmeyer W., Dodds P. (2014). The past, present and future of breeding rust resistant wheat. Front. Plant Sci..

[75] Möller M., Stukenbrock E.H. (2017). Evolution and genome architecture in fungal plant pathogens. Nat. Rev. Genet..

[76] Wellings C.R. (2007). *Puccinia striiformis* in Australia: A review of the incursion, evolution, and adaptation of stripe rust in the period 1979–2006. Aust. J. Agric. Res..

[77] Milus E.A., Kristensen K., Hovmoller M. (2009). Evidence for Increased Aggressiveness in a Recent Widespread Strain of *Puccinia striiformis* f. sp. tritici Causing Stripe Rust of Wheat. Phytopathology.

[78] Hovmoller M., Walter S., Justesen A.F. (2010). Escalating Threat of Wheat Rusts. Science.

[79] Ohm R.A., Feau N., Henrissat B., Schoch C.L., Horwitz B.A., Barry K.W., Condon B.J., Copeland A.C., Dhillon B., Glaser F. (2012). Diverse Lifestyles and Strategies of Plant Pathogenesis Encoded in the Genomes of Eighteen Dothideomycetes Fungi. PLoS Pathog..

[80] Stefansson T.S., McDonald B.A., Willi Y. (2014). The Influence of Genetic Drift and Selection on Quantitative Traits in a Plant Pathogenic Fungus. PLoS ONE.

[81] Ali S., Gladieux P., Leconte M., Gautier A., Justesen A.F., Hovmoller M., Enjalbert J., De Vallavieille-Pope C. (2014). Origin, Migration Routes and Worldwide Population Genetic Structure of the Wheat Yellow Rust Pathogen *Puccinia striiformis* f. sp. tritici. PLoS Pathog..

[82] Kiran K., Ansari S.A., Srivastava R., Lodhi N., Chaturvedi C.P., Sawant S.V., Tuli R. (2006). The TATA-Box Sequence in the Basal Promoter Contributes to Determining Light-Dependent Gene Expression in Plants. Plant Physiol..

