# Supplementary material for: Genome-Wide Analysis of Four Pathotypes of Wheat Rust Pathogen (Puccinia graminis) Reveals Structural Variations and Diversifying Selection
Source: J Fungi (Basel). 2021 Aug 27;7(9):701. doi: 10.3390/jof7090701 (PMC8468629; doi:10.3390/jof7090701)
Supplement: Supplementary file 1 [file jof-07-00701-s001.zip › jof-1318237-supplementary.pdf]

Journal of *Fungi*

Genome wide analysis of four pathotypes of wheat rust pathogen (*Puccinia graminis*) reveals differences at structural variations and diversifying selection

Kanti Kiran[1], Hukam C Rawal[1], Himanshu Dubey[1], Rajdeep Jaswal[1], Subhash C Bhardwaj[2], Rupesh Deshmukh[3] and Tilak Raj Sharma[1,3]

[1] ICAR-National Institute for plant Biotechnology, Pusa Campus New Delhi,
[2] ICAR-Indian Institute of Wheat and Barley Research, Regional Station, Flowerdale, Shimla,
[3] National Agri-Food Biotechnology Institute (NABI), Mohali, Punjab, India -160071
* Correspondence: to: T R Sharma (trsharma1965@gmail.com; ddgcs.icar@nic.in), Phone:91- 11-23382545, FAX: 91-11-23097003

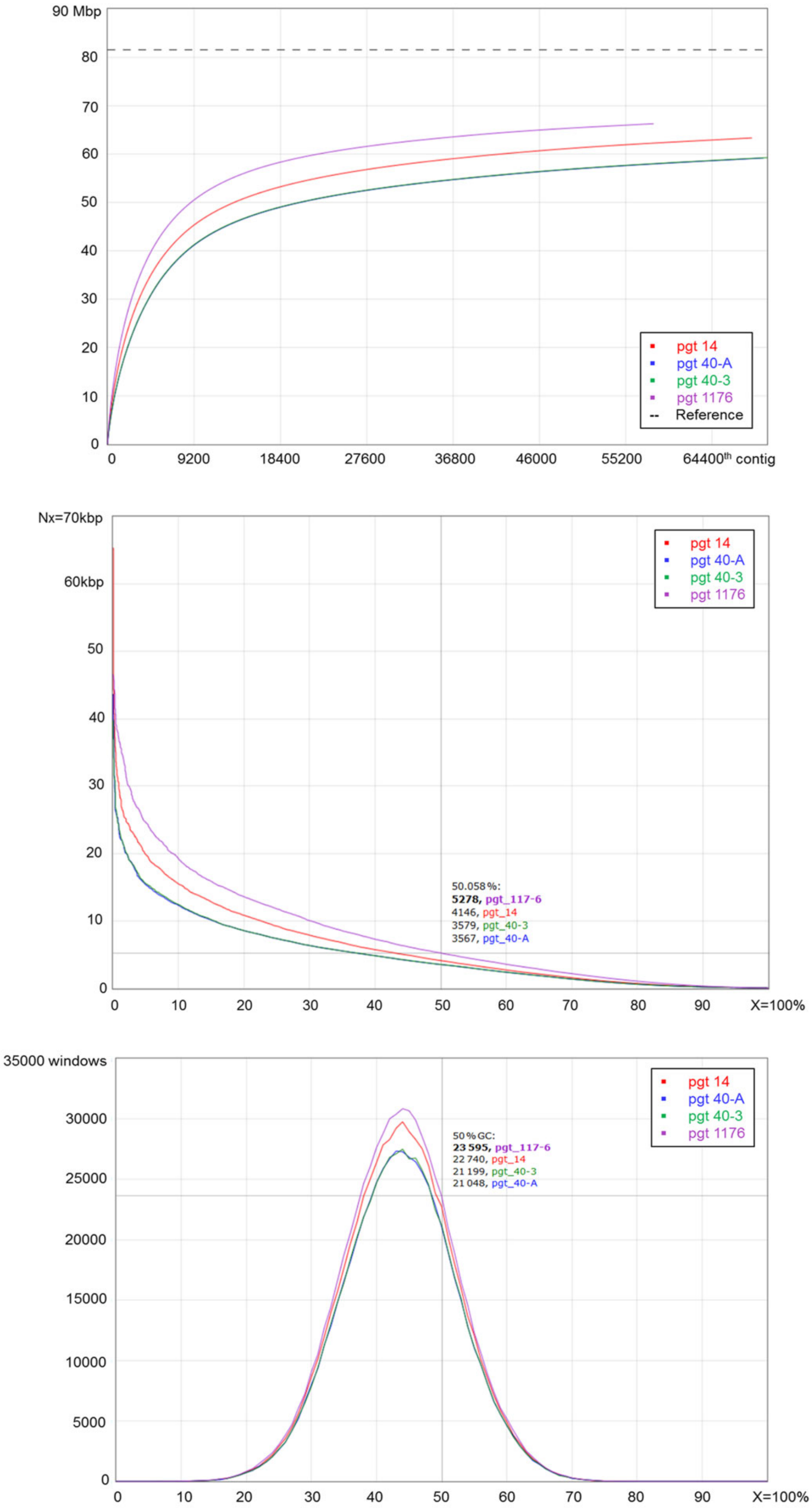

Figure S1. Assembly statistics of *P. graminis* pathotypes. Cumulative length of contig versus the genome size in all the four pathotypes (14, 40A, 40-3 and 117-6), post assembly, contigs are ordered from largest (contig #1) to smallest (**extreme top figure**). Presentation of N50 as a quality of assembly in the three pathotypes (**middle figure**). Whole genome presentations of GC% in the pathotypes, contigs are broken into nonoverlapping 100 bp windows. Plot shows numbers of

windows for each GC percentage (**bottom figure**). Reference used is from Broad Institute *P. graminis* isolate CDL 75-36-700-3, Race SCCL.

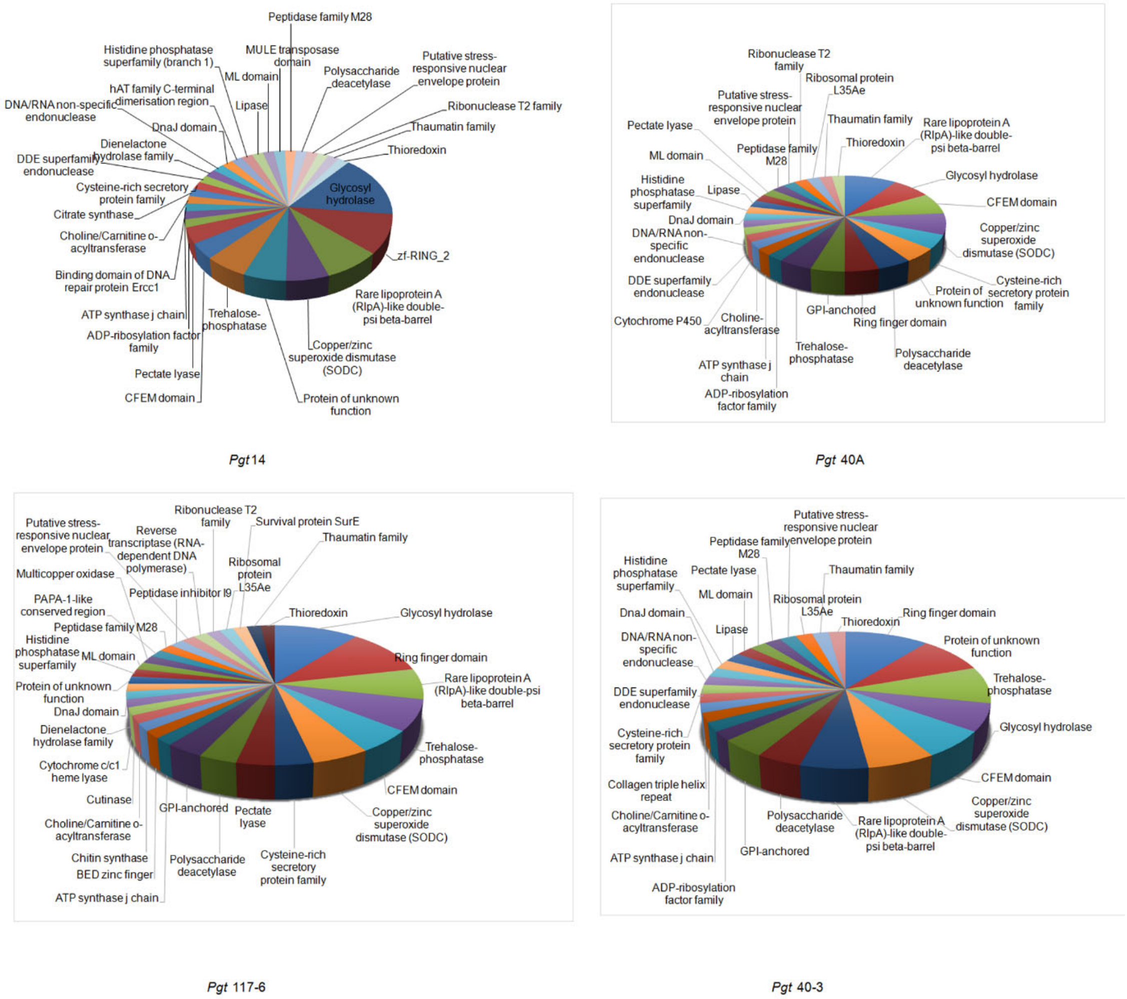

Figure S2. Identification of Conserved domains in the *P. graminis* pathotypes. Conserved domains within the extracellular secretory proteins indentified in the four pathotypes by using Pfam database (pfam.xfam.org/).

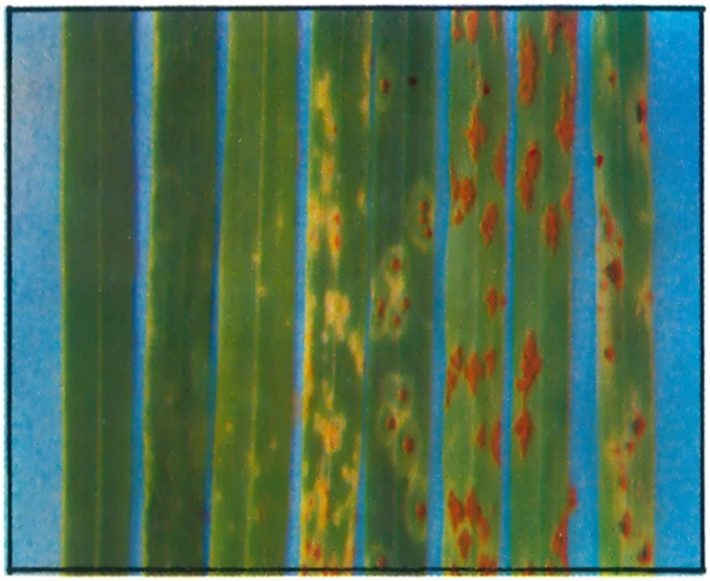

Figure S3. Depiction of IT (infection types) for the code of pathotypes on wheat leaves. Various degree of infection types ranging from 0-3+ achieved by *P.graminis* pathotypes on wheat leaves based on the severity achieved.

Table S1. Genome assembly of the *P. graminis* pathotypes.

**Statistics**	***Pgt* 14**	***Pgt* 40-A**	***Pgt* 40-3**	***Pgt* 117-6**
# contigs	68622	69842	70264	58140
# contigs (>= 1000 bp)	11917	11846	11883	11912
# contigs (>= 5000 bp)	2991	2704	2702	3277
# contigs (>= 10000 bp)	968	657	661	1263
# contigs (>= 25000 bp)	42	16	14	98
# contigs (>= 50000 bp)	1	0	0	0
Largest contig	65288	43631	39779	46544
Total length	63312312	59153492	59251903	66250748
Total length (>= 1000 bp)	48572782	44344139	44461329	53780975
Total length (>= 5000 bp)	28339086	23102244	23159474	34096505
Total length (>= 10000 bp)	14381309	9111835	9223794	20082964
Total length (>= 25000 bp)	1277225	468466	415994	3059556
Total length (>= 50000 bp)	65288	0	0	0
N50	4153	3573	3584	5288
N75	1132	997	1002	1620
L50	3720	4241	4233	3088
L75	10894	11868	11861	8686
GC (%)	43.61	43.65	43.65	43.59
Misassemblies				
# misassemblies	148	157	153	105
# relocations	3	5	4	3
# translocations	145	152	148	102
# inversions	0	0	1	0
# misassembled contigs	140	144	146	97
Misassembled contigs length	62554	50078	51477	39373
# local misassemblies	9	11	12	7
# fully unaligned contigs	26412	30248	30233	17814
Fully unaligned length	6701681	7597323	7599049	3829567

# partially unaligned contigs	4996	5730	5718	3532
# with misassembly	58	61	64	32
# both parts are significant	596	618	601	352
Partially unaligned length	836969	798308	792713	430705
Genome statistics				
Genome fraction (%)	68.12	61.919	62.054	75.834
Duplication ratio	1.004	1.006	1.005	1.003
Largest alignment	65288	43631	39766	46544
NG50	2245	1529	1537	3379

Worst Median Best

*Reference genomnesize: 81521292 bp , G+C content: 43.35 %

Table S2. : Statistics of the completeness of the genome based on analysis by CEGMA.

A. with complete CEGs.

***Pgt* Pathotypes**	**No. of proteins**	**% Completeness**	**Total**	**Average**	**% Ortho**	**Normalized % Completeness**
14	193	77.82	364	1.89	54.92	86.54
40A	188	75.81	394	2.10	63.30	84.30
40-3	188	75.81	393	2.09	63.83	84.30
117-6	198	79.84	365	1.84	52.53	88.79
Reference	223	89.92	307	1.38	28.25	100

B. with partial CEGs

***Pgt* Pathotypes**	**No. of proteins**	**% Completeness**	**Total**	**Average**	**% Ortho**	**Normalized % Completeness**
14	209	84.27	415	1.91	58.85	90.86
40A	205	82.66	446	2.18	64.88	89.13
40-3	205	82.66	436	2.13	63.90	89.13
117-6	212	85.48	400	1.89	53.77	92.17
Reference	230	92.74	343	1.49	34.78	100

Key:

Prots = number of 248 ultra-conserved CEGs present in genome

%Completeness = percentage of 248 ultra-conserved CEGs present

Total = total number of CEGs present including putative orthologs

Average = average number of orthologs per CEG

%Ortho = percentage of detected CEGS that have more than 1 ortholog

Table S3. Indentification of intra-pathotype genetic variation within the *P. graminis* pathotypes.

Mapped Reads	Reference	Total SNPs	SNPs/Kb
Pgt-14	*Pgt*-14	14,98,958	23.66
Pgt -40A	*Pgt*-40A	15,20,994	25.71
Pgt -40-3	*Pgt*-40-3	15,36,330	25.93
Pgt -117-6	*Pgt*-117-6	13,03,490	19.68

Table S4. Identification of inter-pathotypes genetic variations among the *P. graminis* pathotypes.

Mapped Reads	Reference	Total SNPs	Heterokaryotic SNPs/Kb	Homokaryotic SNPs/Kb
Pgt-14	*Pgt*40A	14,92,928	11.31	1.26
Pgt-14	*Pgt*40-3	14,95,107	11.32	1.28
Pgt-14	*Pgt*-117-6	18,74,553	11.99	5.67
Pgt-40A	*Pgt*-14	16,31,882	13.11	1.69
Pgt-40A	*Pgt*-40-3	15,70,240	12.87	1.17
Pgt-40A	*Pgt*-117-6	19,89,425	13.56	6.51
Pgt-40-3	*Pgt*-14	16,45,674	13.20	1.69
Pgt-40-3	*Pgt*-40A	15,81,531	12.95	1.16
Pgt-40-3	*Pgt*-117-6	20,01,892	13.63	6.51
Pgt-117-6	*Pgt*-14	15,45,043	9.39	4.64
Pgt-117-6	*Pgt*-40A	15,62,457	8.99	5.65
Pgt-117-6	*Pgt*-40-3	15,65,119	9.00	5.66

Table S5. Gene predictions of the *P. graminis* pathotypes.

Pathotypes	***Pgt* 14**	***Pgt* 40A**	***Pgt* 117-6**	***Pgt* 40-3**
Genes (≥ 150 bases)	12424	11347	13943	11371
Genes (≥ 450 bases)	9419	8665	10315	8694
Genes with annotation	9250	8507	10141	8547
Genes with annotation (excluding Hypotheticals)	4850	4558	5142	4581

Table S6. Categorization of annotated genes (> 450) into various classes based on their functional roles.

s.no	Functional Class	***Pgt* 14**	***Pgt* 40-A**	***Pgt* 117-6**	***Pgt* 40-3**
	Amino acid biosynthesis	96	93	100	96
2	Biosyn. of cofactors, prosthetic grps& carriers	124	114	124	113
3	Cell envelope	90	82	84	84
4	Cellular processes	332	309	345	312
5	Central intermediary metabolism	42	39	45	43
6	DNA metabolism	203	202	203	207
7	Growth & development	146	134	140	126
8	Energy metabolism	924	875	956	882
9	Fatty acid & phospholipid metabolism	60	58	72	58
10	Hypothetical proteins	4400	3949	4999	3966
11	Mobile &extrachromosomal element functions	354	289	463	300
12	Protein fate	404	380	423	379
13	Protein synthesis	256	252	270	256
14	Purines, pyrimidines, nucleosides & nucleotides	71	68	87	72
15	Regulatory functions	97	100	97	94
16	Signal transduction	69	58	74	60
17	Transcription	267	249	264	243
18	Transport and binding proteins	438	416	438	425
19	Unclassified	136	130	152	123

20	Unknown function	57	61	73	63
21	Conserved domains	319	285	342	294
22	Predicted Protein	363	362	388	349
23	Viral functions	2	2	2	2
	Total	9250	8507	10141	8547

Table S7. Secretome analysis of the *P. graminis* pathotypes.

**Pathotypes**	***Pgt* 14**	***Pgt* 40A**	***Pgt* 40-3**	***Pgt* 117-6**
Total proteins	12424	11347	11371	13934
SignalP	1412	1310	1308	1574
TargetP	2270	2065	2062	2513
SignalP+TargetP merged unique	2299	2094	2098	2551
TmHmm 0 and 1 Tm	1978	1770	1790	2216
ProtComp Extracellular secreted	588	529	535	681

Table S8. Functional Annotation of proteomes in *P. graminis* pathotypes (Against PHI Database).

**Pathotypes**	***Pgt* 14**	***Pgt* 40-A**	***Pgt* 117-6**	***Pgt* 40-3**
Year of Isolation	1959	1974	1990	2008
Genes (≥ 150 bases)	12424	11347	13943	11371
Annotated against PHI	923	874	945	886
% Genes annotated with PHI	7.42	7.70	6.77	7.79

Table S9. Analysis of pairwise orthologous genes across the *P.graminis* pathotypes.

**Orthologous pairs among the pathotypes**				
**Cluster**				**Ortho-Pairs**
Pgt 14-*Pgt*1176				642
Pgt 403-*Pgt* 40A				353
Pgt 14-*Pgt* 40-3				160
P.gr 14- *Pgt* 40A				146
Pgt 1176- *Pgt* 40A				127
Pgt 403- *Pgt* 1176				122
Pgt 14- *Pgt* 403- *Pgt* 1176				308
Pgt 14- *Pgt* 40A- *Pgt* 1176				271
Pgt 403- *Pgt* 40A- *Pgt* 1176				234
Pgt 403- *Pgt* 40A- *Pgt* 14				622
Pgt 40A- *Pgt* 14- *Pgt* 403- *Pgt* 1176				7106
**Specificity and unique features of the four pathotypes**				
P. graminis pathotypes	14	40A	40-3	117-6
Genes having orthologs (across species)	8580	8072	8126	8340
Pure paralogous genes with no orthologs across species	9	0	1	93
Genes with neither ortho- nor paralogs	830	593	567	1882

Table S10. Diversifying selection analysis of genes (≥450bp) in four *P.graminis* pathotypes.

**Genome**	**Genes (≥450bp)**	**Genes analyzed by YN00**	**Mean *dN/dS* ratio**	**Genes analyzed by CODEML**
Pgt 117-6	10315	8340 (80.85%)	0.32	415 (4.02%)
Pgt 14	9419	8580 (91.09%)	0.28	440 (4.67%)
Pgt 40-3	8694	8072 (92.85%)	0.28	430 (4.95%)
Pgt 40A	8665	8126 (93.78%)	0.28	435 (5.02%)

Table S11. Genes under specific selection pressure in the *P. graminis* pathotypes.

**Genome**	**YN00 based Analysis**			**CODEML (Site-specific diversifying genes) (P <0.05)**	
	**Genes with (*dN/dS*) = 1**	**Genes with (*dN/dS*) < 1**	**Genes with (*dN/dS*) > 1**	**Purifying Selection ((*dN/dS*) < 1)**	**Positive Selection ((*dN/dS*) > 1)**
Pgt 117-6	1	8073 (96.80%)	266 (3.19%)	155 (37.35%)	119 (28.67%)
Pgt 14	0	8336 (97.16%)	244 (2.84%)	148 (33.64%)	137 (31.14%)
Pgt 40-A	1	7841 (97.14%)	230 (2.85%)	155 (36.05%)	125 (29.07%)
Pgt 40-3	0	7887 (97.06%)	239 (2.94%)	150 (34.48%)	132 (30.34 %)

Table S12. Functional classification of genes with omega (dN/dS) > 1.

**s.no**	**Functional Class**	***Pgt* 117-6**	***Pgt* 14**	***Pgt* 40A**	***Pgt* 40-3**
1	Amino acid biosynthesis	3	1	0	1
2	Biosyn. of cofactors, prosthetic grps& carriers	1	1	0	0
3	Cell envelope	0	0	0	0
4	Cellular processes	4	6	3	5
5	Central intermediary metabolism	1	1	1	2
6	DNA metabolism	1	0	1	0
7	Growth & development	0	2	1	2
8	Energy metabolism	8	8	11	7
9	Fatty acid & phospholipid metabolism	0	0	0	0
10	Hypothetical proteins	202	184	174	179
11	Mobile &extrachromosomal element functions	10	7	7	5
12	Protein fate	6	6	4	6
13	Protein synthesis	2	3	3	2
14	Purines, pyrimidines, nucleosides & nucleotides	0	0	1	0
15	Regulatory functions	1	1	2	1
16	Signal transduction	0	0	1	1
17	Transcription	0	0	1	2
18	Transport and binding proteins	5	1	2	5
19	Unclassified	2	3	2	1
20	Unknown function	0	3	2	2
21	Conserved domains	4	6	4	4
22	Predicted Protein	9	3	3	5
23	Viral functions	0	0	0	0
24	Un-annotated	8	8	8	9
	Total	267	244	231	239

Table S13. Functional classification of Site-specific diversifying genes.

s.no	Functional Class	*Pgt* 117-6	*Pgt* 14	*Pgt* 40-A	*Pgt* 40-3
1	Amino acid biosynthesis	3	1	0	1
2	Biosyn. of cofactors, prosthetic grps & carriers	1	1	0	0
3	Cell envelope	0	0	0	0
4	Cellular processes	2	5	1	5
5	Central intermediary metabolism	0	0	0	2
6	DNA metabolism	0	0	1	0
7	Growth & development	0	1	1	2
8	Energy metabolism	4	5	9	4
9	Fatty acid & phospholipid metabolism	0	0	0	0
10	Hypothetical proteins	83	100	86	91
11	Mobile &extrachromosomal element functions	5	4	5	4
12	Protein fate	1	4	3	3
13	Protein synthesis	1	2	1	0
14	Purines, pyrimidines, nucleosides & nucleotides	0	0	1	0
15	Regulatory functions	1	1	2	1
16	Signal transduction	0	0	1	1
17	Transcription	0	0	1	1
18	Transport and binding proteins	4	0	2	3
19	Unclassified	2	2	2	1
20	Unknown function	0	2	2	1
21	Conserved domains	3	4	2	4
22	Predicted Protein	4	3	2	4
23	Viral functions	0	0	0	0
	Un-annotated	5	2	3	4
	Total	119	137	125	132

Table S14. Virulence /avirulence formula for the *P. graminis* pathotypes.

Pgt pathotypes	Avirulence genes	Virulence genes
**14**	*Sr* 5, *Sr* 7a, *Sr* 9b, *Sr* 9e, *Sr*11, *Sr* 13, *Sr* 17, *Sr* 22, *Sr* 23, *Sr*24, *Sr* 25, *Sr* 26, *Sr* 27, *Sr* 28, *Sr* 29, *Sr* 30, *Sr* 31,*Sr* 32, *Sr* 33, *Sr* 34, *Sr* 35, *Sr* 36,*Sr* 37, *Sr* 39, *Sr* 40, *Sr* 43, *Sr* Tmp, *Sr* Tt3	*Sr* 2, *Sr*6, *Sr*7b, *Sr* 8a, *Sr* 8b, *Sr* 9a, *Sr* 9d, *Sr* 9f, *Sr* 9g, *Sr* 10, *Sr* 14, *Sr* 15, *Sr* 16, *Sr* 18, *Sr* 19, *Sr* 20, *Sr* 21, *Sr* McN
**40A**	*Sr* 7a, *Sr* 13, *Sr* 21, *Sr* 22, *Sr*24, *Sr* 25, *Sr* 26, *Sr* 27, *Sr* 30, *Sr* 31, *Sr* 32,*Sr* 33, *Sr* 35, *Sr* 36, *Sr* 37, *Sr* 39, *Sr* 40, *Sr* 43, *Sr* Tmp, *Sr* Tt3	*Sr* 2, *Sr* 5, *Sr*6, *Sr*7b, *Sr* 8a, *Sr* 8b, *Sr* 9a, *Sr* 9b, *Sr* 9d, *Sr* 9e, *Sr* 9f, *Sr* 9g, *Sr* 10, *Sr* 14, *Sr* 15, *Sr* 16, *Sr* 17, *Sr* 18, *Sr* 19, *Sr* 20, *Sr* 23, *Sr* 28, *Sr* 29, *Sr* 34,*Sr* McN
**40-3**	*Sr* 21, *Sr* 22,*Sr*24, *Sr* 25, *Sr* 26, *Sr* 27, *Sr* 31, *Sr* 32, *Sr* 33, *Sr* 35, *Sr* 36, *Sr* 37, *Sr* 39, *Sr* 40, *Sr* 42, *Sr* 43, *Sr* Tmp, *Sr* Tt3	*Sr* 2, *Sr* 5, *Sr* 6, *Sr* 7a, *Sr* 7b, *Sr* 8a,*Sr* 8b, *Sr* 9a, *Sr* 9b, *Sr* 9d, *Sr* 9e, *Sr* 9f, *Sr* 9g, *Sr* 10, *Sr* 11, *Sr* 13, *Sr* 14, *Sr* 15, *Sr* 16, *Sr* 17, *Sr* 18, *Sr* 19, *Sr* 20, *Sr* 23, *Sr* 28, *Sr* 29, *Sr* 30, *Sr* 34, *Sr* 38, *Sr* 44, *Sr* McN, *Sr* Gt
**117-6**	*Sr*5, *Sr* 7a, *Sr*8a, *Sr*8b, *Sr*9b, *Sr*11,*Sr* 12 *Sr*22, *Sr*24, *Sr* 25, *Sr* 26, *Sr* 27, *Sr* 28, *Sr* 30, *Sr* 31, *Sr* 32, *Sr* 33, *Sr* 35, *Sr* 36, *Sr* 37, *Sr* Tmp	*Sr* 2, *Sr* 6, *Sr* 7a, *Sr* 7b, *Sr* 9e, *Sr* 9f,*Sr* 9g, *Sr* 10, *Sr* 11, *Sr* 12, *Sr* 13, *Sr* 14, *Sr* 15, *Sr* 16, *Sr* 17, *Sr* 19, *Sr* 21, *Sr* 23, *Sr* 29, *Sr* 34,*Sr* McN

Table S15. wheat lines/variety of wheat carrying *Sr* genes.

Set 0 (Near isogenic wheat lines/variety)	Set A (Near isogenic wheat lines/variety)	Set B (wheat Variety)
Sr24 (3D/Ag Translocations)	*Sr13* (Khapstein/10 Marquis)	Marquis (*Sr7b*+*Sr18*+*Sr19*+*Sr20*)
NI 5439 (*Sr11*)	*Sr9b* (CS/KF 2B)	Einkorn (*Sr21*)
Sr25 (7D/Ag translocations)	*Sr11* (I*Sr11*Ra= W 3015)	Kota (*Sr28*+ *Sr7b*)
DWR195(*Sr2*+*Sr31*)	*Sr28* {Kota ; = *Sr28* (CH 71)}	Reliance (*Sr5*+ *Sr16*+*Sr18*+*Sr20*)
HD2189(*Sr2*+*Sr31*)	*Sr8b* (I*Sr8a*Ra = W3384)	Charter (*Sr11*+)
LoK 1(*Sr2*+*Sr9b*+*Sr11*)	*Sr9e* (Vernstein)	Khapli (*Sr7a, Sr13, Sr14*)
HI1077(*Sr2*+*Sr9b*+*Sr11*)	*Sr30* (Festiguay)	Tc*6/*Sr31*/*Lr26*(*Yr9*)
Barley Local (Susceptible barley check)	*Sr37* (Line W)	--
Agra Local (Susceptible bread wheat check)	--	--

Table S16. Segmental Duplication in *P. graminis* genomes.

***Pgt* Pathotypes**	**14**	**40A**	**117-6**	**40-3**
**Assembled Genome Size (Mb)**	63.31	59.15	66.25	59.25
**Segmental Duplication (Mb)**	0.38	0.26	0.76	0.24
**SDs % in Genome**	0.60	0.44	1.14	0.40
**SDs (≥95% Identity) (Mb)**	0.023	0.012	0.091	0.014
